# Extending Thioflavin T Fluorescence Probe to 2‑Ethenyl-benzothiazole Derivatives: Drug-like Quadruplex Ligands with Potent Antitrypanosomatid Activity

**DOI:** 10.1021/acsinfecdis.5c00691

**Published:** 2025-11-04

**Authors:** Raquel C. R. Gonçalves, Pablo Peñalver, Nina M. Allen, Efres Belmonte-Reche, Belén García-Pérez, Susana P. G. Costa, Y. Jennifer Jiang, José María Pérez-Victoria, M. Carmen Galan, M. Manuela M. Raposo, Juan Carlos Morales

**Affiliations:** † Centre of Chemistry, 56059 University of Minho, Campus of Gualtar, 4710-057 Braga, Portugal; ‡ Departamento de Biología y Farmacología Molecular, 38800 Instituto de Parasitología y Biomedicina López Neyra, CSIC, PTS Granada, Avenida del Conocimiento, 17, Armilla, 18016 Granada, Spain; § School of Chemistry, 1980 University of Bristol, Bristol BS8 1TS, U.K.; ∥ Centre for Genomics and Oncological Research, Pfizer/University of Granada/Andalusian Regional Government, 421117 GENYO, PTS Granada, Av. de la Ilustración, 114, 18016 Granada, Spain; ⊥ Department of Biochemistry and Molecular Biology II, Faculty of Pharmacy, University of Granada, 18071 Granada, Spain; # Hospital Virgen de las Nieves, Instituto de Investigación Biosanitaria ibs.GRANADA, 18014 Granada, Spain; a Advanced (Magnetic) Theranostic Nanostructures Lab, International Iberian Nanotechnology Laboratory, 4715-330 Braga, Portugal

**Keywords:** leishmaniasis, African trypanosomiasis, antiparasitic agents, benzothiazolium derivatives, G-quadruplex ligands

## Abstract

Thioflavin T (ThT) is a well-established fluorescence probe with selectivity for G-quadruplex (G4) structures. Over the past few years, G4 ligands have emerged as promising candidates for the development of antiparasitic agents. Building on this concept, we explored extending ThT’s benzothiazole scaffold by introducing various 2-ethenyl aromatic and heteroaromatic moieties, aiming to enhance G4 binding affinity and potential therapeutic effect. A series of benzothiazolium derivatives were synthesized and evaluated for their antiproliferative and antiparasitic activity. Several 2-ethenyl benzothiazole derivatives showed submicromolar activity against *Leishmania* spp. and *Trypanosoma brucei* parasites, with up to 200-fold selectivity over MRC-5 human lung fibroblasts. Notably, compound **2b** demonstrated remarkable potency, with an IC_50_ of 0.48 nM and a selectivity index of 46,151 against *Leishmania major* amastigotes, and an IC_50_ of 0.019 nM and a selectivity index of 79,206 against *T. brucei*. In fact, compound **2b** demonstrated superior efficacy and selectivity in comparison to the clinically used drugs suramin, fexinidazole, miltefosine, and amphotericin B. Biophysical studies revealed that all tested derivatives exhibited significant G4 stabilization, surpassing ThT. Location of compound **2b** inside the nucleus and the kinetoplast, as well as partially in the mitochondria, opens up the possibility of **2b** acting against the parasite through binding to G4.

## Graphical abstract

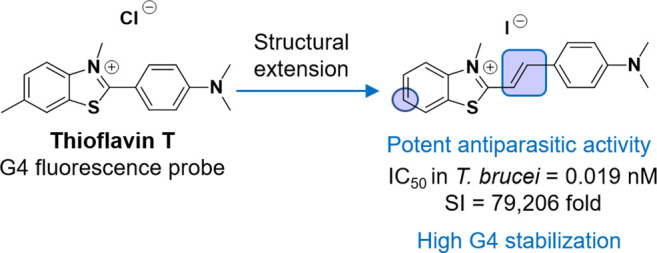

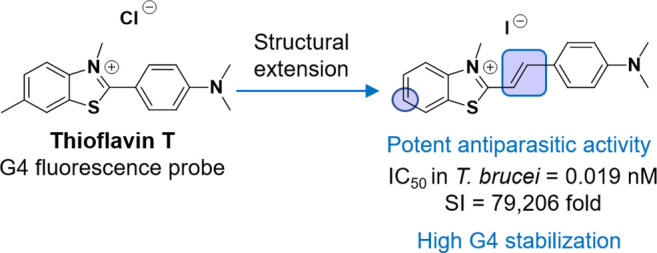

G-Quadruplexes (G4), unique secondary structures formed by guanine-rich DNA and RNA sequences, have attracted considerable attention due to evidence of their implication in many cellular and genetic processes. They are assembled by stacked planar arrangements of four guanine bases (G-tetrads) that are held together by Hoogsteen hydrogen bonds. The stability of these structures is particularly dependent on the interaction with monovalent cations, especially Na^+^ and K^+^, and small molecules, known as G4 ligands.^1,2^ Computational methods have been developed to predict putative quadruplex sequences (PQS) in genomic sequences. These methods employ models to analyze the sequence properties and structural features associated with G4 formation.^3^ Through biophysical techniques such as nuclear magnetic resonance (NMR), X-ray crystallography, circular dichroism spectroscopy, Förster resonance energy transfer (FRET), and UV melting, the G4-forming capacity of specific sequences can be validated. Furthermore, chromatin-immunoprecipitation sequencing (ChIP-seq)-based mapping methods have been described for the identification and characterization of G4 structures at a genome-wide level, thus providing evidence for the existence of G4 in vitro*.* ^4–6^

Putative G4 motifs have been identified across the genomes of many species, including mammals, bacteria, viruses, parasite, and so forth.^7^ Interestingly, specific genomic regions, such as promoters, enhancers, telomeres, and untranslated regions (UTRs) of mRNA, are particularly rich in G4-forming sequences.^2,8,9^ In fact, G4 structures have been proven to display functional roles as regulatory elements in gene expression, in DNA replication, in telomere maintenance, and in genome stability.^2,10–13^ G4 motifs have been explored as targets in cancer therapy due to their prevalence in tumor-related gene promoters, such as KRAS, c-MYC, c-KIT, and BCL-2,^14^ and small molecules that selectively bind and stabilize these structures have shown potential as anticancer drugs.^15^ In a similar way, the identification of G4 structures in the genome of pathogenic protozoa has encouraged the development of G4 ligands with promising antiparasitic activity.^16,17^

Leishmaniasis, caused by *Leishmania* spp., and human African trypanosomiasis (or sleeping sickness), caused by *Trypanosoma brucei* (*gambiense* and *rhodesiense* subspecies), are neglected tropical diseases (NTDs) that have been recently proposed in the NTD roadmap for 2021–2030 by the World Health Organization, with the goal of enhancing efforts to prevent and manage these protozoan infections.^18^ The treatment of these neglected tropical diseases is often challenging due to the significant limitations of existing antiprotozoal drugs including severe side effects, the emergence of drug resistance, and high costs. Thus, it is of great importance to develop effective antiparasitic agents. Recently, the role of G4s as transcriptional regulators in trypanosomatid parasites, namely, *Trypanosoma* and *Leishmania* spp., has been reviewed.^16^ Studies revealed that G4 motifs could have significant regulatory functions in these protozoan pathogens, particularly as modulators of kinetoplastid DNA replication, as transcriptional regulators of epigenetic modifications, and in antigenic variation. Hence, these findings further support the idea that G4-targeting molecules may be a promising therapeutic strategy. In the last years, compounds based on naphthalene diimide, perylene diimide, stiff-stilbene, phenanthroline, quinazoline, diquinolinyl-pyridine, azobenzene, and dithienylethene cores have been reported as G4 binders that display potent antiparasitic activity.^19–27^

Thioflavin T (ThT), a benzothiazole derivative (Figure 1), was first reported as a fluorescent probe for the detection of amyloid fibrils.^28^ Decades later, Mohanty et al. demonstrated the dual role of ThT as a quadruplex folding-inducer in the 22AG human telomeric DNA and as a specific G4-fluorescence probe through an emission enhancement upon interaction with quadruplex motifs.^29^ Moreover, ThT exhibited distinct recognition of RNA G-quadruplex structures in contrast to other forms of RNA, such as single-stranded RNA (ss-RNA) and hairpin RNA.^30^ Other benzothiazole-based probes have been studied for the fluorescence detection of G-quadruplex structures.^31–33^

**Figure 1:**
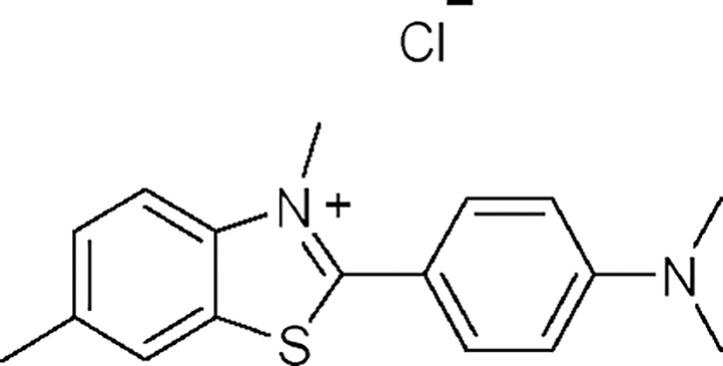
Chemical structure of thioflavin T (ThT).

The benzothiazole core is an important scaffold with a wide range of pharmacological properties including anticancer, anti-inflammatory, antidiabetic, anticonvulsant, antioxidant, antiviral, and antimicrobial activity.^34^ The antiparasitic activity of derivatives based on this scaffold has also been described.^35–43^ Push–pull benzothiazole structures, with an electron-acceptor benzothiazolium moiety functionalized with electron-donor amino-substituted benzene groups through a π-conjugated bridge, have been associated with higher antimicrobial activity.^44,45^ More recently, Wu et al. reported a new family of benzothiazole derivatives as potential G4 ligand-based c-*MYC* transcription inhibitors for cancer treatment.^46^

In this work, we explored extending the benzothiazole scaffold of ThT with varied 2-ethenyl aromatic and heteroaromatic groups. Our plan was to investigate how different C-2 modifications of the benzothiazolium core affect their G4 binding capability and their therapeutic potential as antiproliferative and antiparasitic agents. The derivatives were evaluated in tumor cell lines (HeLa and HT-29) and nontumor cell lines (MRC-5 and THP-1), and in the parasites *Leishmania major* extracellular promastigotes and intracellular amastigotes, the clinically relevant form and in bloodstream forms of *Trypanosoma brucei* parasites. The intracellular localization and in silico ADMET profile of the best compound were also studied. Finally, we confirmed the G4 binding ability of these compounds using a variety of biophysical techniques.

## Results and Discussion

### Synthesis and Characterization of 2-Ethenyl Benzothiazolium Derivatives

The new 2-ethenyl benzothiazolium derivatives **2d**, **2f**, **2i**, and **2j** were synthesized through the Knoevenagel condensation reaction of 2,3-dimethylbenzo­[*d*]­thiazol-3-ium iodide with the corresponding aldehydes **1d**, **1f**, **1i**, and **1j** under reflux in ethanol. All compounds precipitated from the reaction mixture and were washed with cold ethanol to give the pure benzothiazolium derivatives in moderate to low yields (Table S1). The yields obtained were low (8–30%), most probably due to the reduced reactivity of the corresponding aldehydes, which likely hindered the progress of the Knoevenagel condensation, and the difficulty in isolating the final products. In both cases, unreacted starting materials were the predominant components, suggesting incomplete conversion rather than the formation of major side products.

Experimental details and structure characterization data are described in the Supporting Information. The synthesis of benzothiazolium derivatives **2a**, **2b**, **2c**, **2e**, **2g**, and **2h** has been described previously by our research group and others.^47–49^ The synthesis and photophysical characterization are presented in Scheme 1 and Table S1.

**Scheme 1:**
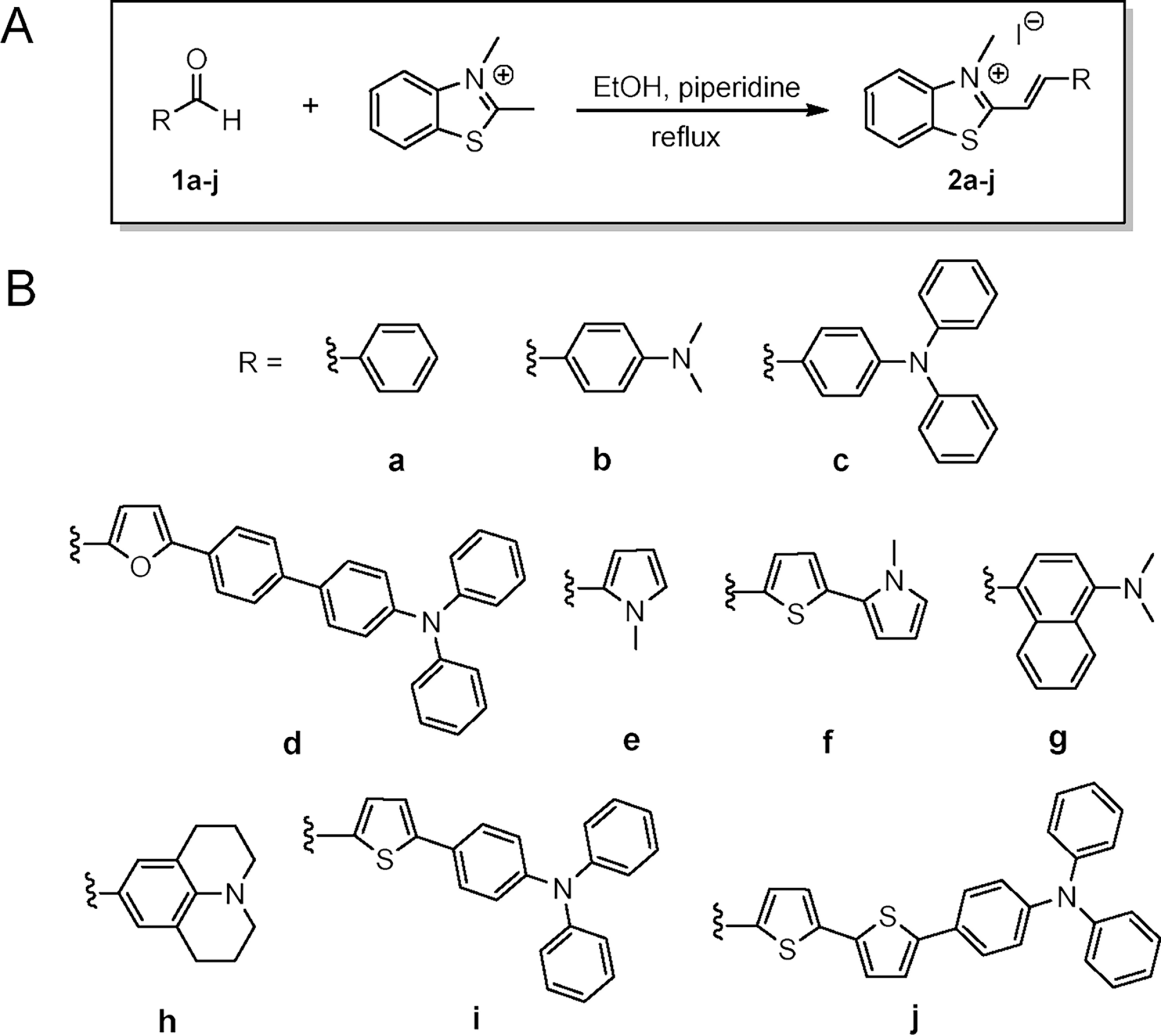
(A) Synthesis of 2-Ethenyl Benzothiazolium Derivatives **2a**–**j**; (B) Structures of the Compounds Prepared

### Antiparasitic and Anticancer Activity

To investigate the biological activity of the benzothiazolium compounds **2a**–**j**, their toxicity was assessed in the bloodstream form of *Trypanosoma brucei* and promastigote and amastigote forms of *L. major* parasites. The antiparasitic activity of ThT, TMPyP4, and RHPS4 (G-quadruplex ligand references), suramin and fexinidazole (*T. brucei* positive drug controls), and miltefosine and amphotericin B (*L. major* positive drug controls) was also included. The antiproliferative activity was investigated in cervical carcinoma (HeLa) and colorectal adenocarcinoma (HT-29) human cancer cell lines. Cytotoxicity was evaluated against control human lung fibroblast (MRC-5) and human leukemia monocytic (THP-1) cell lines. Cell viability results were expressed as the half-maximal inhibitory concentration (IC_50_) calculated through dose–response curves, and the selectivity index (SI) was determined by the ratio of IC_50_ in healthy cells to IC_50_ in parasites or cancer cells, in order to predict the potential therapeutic window of each compound (Tables 1–3).

**Table 1:** Antiparasitic Activity against Bloodstream Form of *T. brucei* and Promastigotes Form of *L. major* and Cytotoxicity in MRC-5, HeLa and HT-29 Represented as IC_50_ Values (μM) with Error Presented as σ^a^

	IC_50_ (μM)					selectivity index (SI)^b^			
compound	*T. brucei*	*L. major*	MRC-5	HeLa	HT-29	*T. brucei*	*L. major*	HeLa	HT-29
**2a**	0.37 ± 0.18	0.48 ± 0.11	2.02 ± 0.12			5.5	4.2		
**2b**	**0.000019 ± 0.0000053**	**0.02 ± 0.013**	1.52 ± 0.37	9.28 ± 0.77	6.56 ± 0.49	**79,206.5**	62.7	0.16	0.23
**2c**	0.34 ± 0.13	**0.02 ± 0.011**	5.13 ± 0.76	8.89 ± 1.59	8.91 ± 0.88	15.1	**252.0**	0.58	0.58
**2d**	**0.01 ± 0.005**	0.33 ± 0.017	3.24 ± 0.14	5.28 ± 0.11	3.79 ± 0.13	**324.5**	9.8	0.62	0.86
**2e**	2.29 ± 0.31	0.24 ± 0.029	9.40 ± 1.06	78.01 ± 8.26	76.42 ± 1.44	4.1	39.6	0.12	0.12
**2f**	0.61 ± 0.045	0.61 ± 0.092	3.68 ± 0.17			6.0	6.0		
**2g**	**0.02 ± 0.015**	0.17 ± 0.04	4.01 ± 0.09	29.08 ± 1.05	89.49 ± 9.55	**200.3**	23.9	0.14	0.04
**2h**	**0.00026 ± 0.00017**	**0.0065 ± 0.0029**	0.08 ± 0.03	0.84 ± 0.01	1.00 ± 0.16	**294.5**	12.0	0.09	0.08
**2i**	1.83 ± 0.06	0.045 ± 0.012	3.88 ± 0.17			2.12	86.2		
**2j**	0.37 ± 0.04	0.99 ± 0.11	4.00 ± 0.14			10.81	4.0		
**ThT**	**0.0047 ± 0.0003**	0.023 ± 0.0002	0.183 ± 0.038			38.9	7.9		
**TMPyP4**	>10^c^	20.82 ± 4.86^c^	>25^c^				>1.7^c^		
**RHPS4**	0.35 ± 0.06	0.044 ± 0.015	4.12 ± 0.52			11.77	93.64		
**suramin**	0.0042 ± 0.0003								
**fexinidazole**	0.88 ± 0.02								
**miltefosine**		5.71 ± 0.46							
**AmB**		1.92 ± 0.05							

a The selectivity index was calculated to be related to a healthy cell line (MRC-5). AmB is Amphotericin B.

b SI = IC_50_ MRC-5/IC_50_ parasite or cancer cell line.

c From ref 19.

All tested compounds exhibited potent antitrypanosomal activity, with IC_50_ ranging five-orders of magnitude (from 0.000019 to 2.39 μM). Compounds **2b**, **2d**, **2g**, and **2h** were notably active as well as selective against *T. brucei*, with IC_50_ in the nanomolar range and SI above 200-fold. These compounds were considerably more active than the reference drug fexinidazole (IC_50_ = 0.88 μM). TMPyP4 and RHPS4, well-established G4 ligands known for their telomerase inhibitory activity in cancer cells,^50,51^ exhibited IC_50_ values >10 and 0.35 μM, respectively, both higher than those observed for the most active benzothiazolium derivatives. Importantly, the derivative functionalized with an (*N,N*-dimethylamino)­phenyl moiety (**2b**) was particularly active, displaying the lowest IC_50_ (0.019 nM) together with a remarkable 79,206-fold selectivity toward the parasites. The toxicity and therapeutic window of this compound are substantially higher than other recently reported antitrypanosomal agents,^21,23^ as well as currently approved clinical drugs. We were also interested in investigating the antiparasitic activity of ThT, a selective G4-fluorescence probe, due to its structural similarity to our derivative **2b**. We observed that the absence of the ethenyl bridge and the C-6 substitution of the benzothiazole core drastically decreased the toxicity for *T. brucei* compared with compound **2b** (IC_50_ = 4.7 nM vs 0.019 nM, respectively). Moreover, ThT displayed higher toxicity on MRC-5 cells, resulting in lower selectivity compared with that of compound **2b.**

When we compared compound **2c** with **2d**, **2i**, and **2j**, we observed that introducing a linker between the ethenyl-benzothiazolium scaffold and the triphenylamine group affected the activity and selectivity. Introducing a 5-phenylfuran linker (compound **2d**) considerably increased activity and selectivity, while replacing it with a thiophene (compound **2i**) or 2,2′-bithiophene (compound **2j**) moiety did not show improvements.

Given the exceptionally high antitrypanosomal activity of **2b** (IC_50_ in the low nM range), the CellTiter-Glo luminescent cell viability assay was used to further corroborate our results (Table 2). The IC_50_ values obtained with this assay (0.00055 μM) were consistent with those obtained through the resazurin methodology. Although the IC_50_ was 27-fold higher when measured by the CellTiter-Glo luminescent method, this difference is not considered significant within the variability typically associated with distinct biological assays. Overall, compound **2b** demonstrated significantly greater activity and selectivity compared to those of our reference and the parent compound, ThT.

**Table 2:** Antiparasitic Activity against *T. brucei* Was Measured by the CellTiter-Glo® Assay

	IC_50_ (μM)		selectivity index (SI)^a^
compounds	*T. brucei*	MRC-5	*T. brucei*
**2b**	0.00055 ± 0.00023	1.52 ± 0.37	2,763.6
**ThT**	0.062 ± 0.003	0.183 ± 0.038	2.95

a SI = IC_50_ MRC-5/IC_50_ *T. brucei*.

Concerning antileishmanial activity, the tested compounds displayed significantly higher potency (IC_50_ ranging from 0.0065 to 0.99 μM) than the reference drug miltefosine (IC_50_ = 5.71 μM) and amphotericin B (IC_50_ = 1.92 μM), as well as than the G4 ligand TMPyP4 (IC_50_ = 20.82 μM). While RHPS4 (IC_50_ = 0.044 μM) showed higher potency than some of the tested compounds, the majority of the benzothiazolium derivatives displayed comparable or superior activity. Compound **2h** was the most toxic against the parasites, with an IC_50_ of 0.0065 μM; however, the derivative **2c**, substituted with a triphenylamino group, exhibited lower toxicity toward MRC-5 cell lines, resulting in a selectivity index 21-fold higher than compound **2h** (SI = 252 vs 12, respectively). Furthermore, introducing different linkers between the ethenyl-benzothiazolium unit and the triphenylamine moieties generally reduced selectivity and, in most cases, also decreased toxicity. For instance, compound **2d** featuring a 5-phenyl-furan group, and compound **2j**, featuring a 2,2′-bithiophene group, both exhibited reduced toxicity and selectivity compared to compound **2c**. Conversely, the introduction of a thiophene heterocycle (compound **2i**) resulted in increased toxicity while still exhibiting selectivity lower than that of compound **2c**. Moreover, comparing the antileishmanial activity of ThT with the synthesized compounds, it was observed that although it exhibited similar activity as compound **2c**, the selectivity toward MRC-5 cells was considerably lower. Overall, compound **2b** demonstrated the greatest therapeutic potential for bloodstream forms of *T. brucei*, while compound **2c** displayed the best potential for promastigotes forms of *L. major*.

Further experiments were conducted to evaluate the activity of compounds **2b** and **2c** against the intracellular amastigote form of *L. major* (Table 3). Interestingly, compound **2b** demonstrated significantly greater potency against the amastigotes compared to the promastigote form (IC_50_ = 0.000484 μM vs 0.02 μM, respectively). Moreover, it exhibited a remarkable selectivity index over THP-1 macrophages (SI = 46,151) and a 50-fold increase in selectivity toward MRC-5 cells when tested against the intracellular form of the parasite. In contrast, the activity of compound **2c** was similar against both promastigote and amastigote forms of the parasite; however, its selectivity index was lower for THP-1 macrophages compared to MRC-5 cells (SI = 33 vs 182, respectively). Moreover, compound **2b** demonstrated a 62-fold increase in activity compared to the G4 ligand RHPS4 and approximately 10,000-fold greater potency than the clinically used drugs miltefosine and amphotericin B, along with a substantially higher selectivity index.

**Table 3:** Antileishmanial Activity against Amastigotes Form of *L. major*

	IC_50_ (μM)			**selectivity index (SI)** ^a^	
compound	*L. major*	THP-1	MRC-5	THP-1	MRC-5
**2b**	**0.000484 ± 0.000617**	**0.48 ± 0.11**	1.52 ± 0.37	**46,151**	**3,132**
**2c**	0.0281 ± 0.00473	0.93 ± 0.02	5.13 ± 0.76	33	182
**RHPS4**	0.03 ± 0.02	213.93 ± 3.36	4.12 ± 0.52	7,131	137.33
**miltefosine**	5.1 ± 0.56	55.19 ± 0.11		10.82	
**AmB**	4.41 ± 1.96	9.89 ± 1.98		2.24	

a SI = IC_50_ THP-1/IC_50_ *L. major* or IC_50_ MRC-5/IC_50_ *L. major*.

The anticancer activity of the derivatives **2b**, **2c**, **2d**, **2e**, **2g**, and **2h** was evaluated. However, all the compounds demonstrated lower activity compared to the activity against the parasites and poor selectivity for the cancer cells over the noncancerous MRC-5 cell line (SI < 1).

### Subcellular Localization of Derivative **2b**

The uptake and subcellular localization of **2b** in MRC-5 cells and *T. brucei* parasites were investigated through fluorescence microscopy, taking advantage of the intrinsic fluorescence of **2b**. After 30 min of incubation, the compound was internalized into both types of cells, exhibiting a nonspecific intracellular distribution, with fluorescence detected in both the cytoplasm and mitochondria (Figures 2A and 3A). At longer periods of incubation, the fluorescence of compound **2b** showed increased colocalization in the nucleus and kinetoplast, suggesting that the compound specifically localizes in these organelles over time (Figures 2B and 3B,C). These findings are particularly significant, as DNA G-quadruplexes mainly form in the nucleus and kinetoplast, and mitochondria, emphasizing the importance of the ability of **2b** to reach and accumulate within these specific organelles.

**Figure 2:**
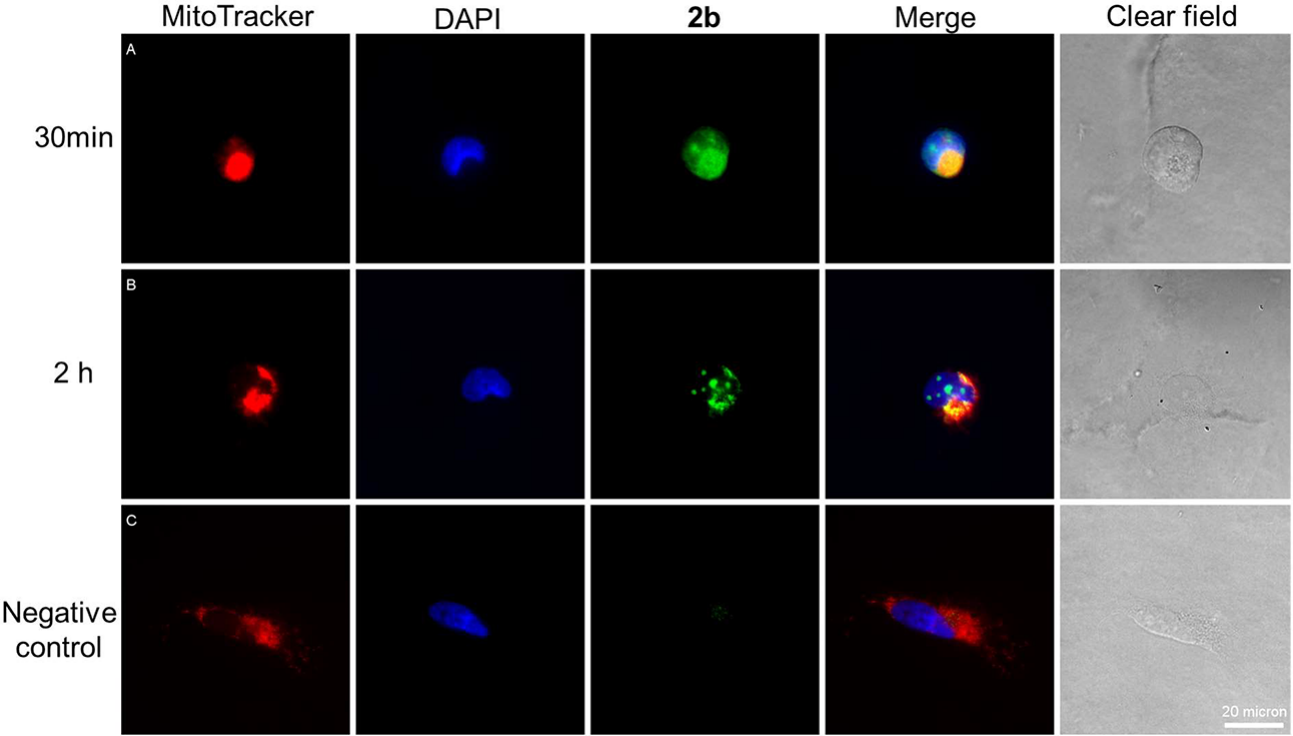
Fluorescence microscopy of MRC-5 cells treated with compound 2b. Cells were incubated with 5 μM compound **2b** (green channel) for 30 min (A) and 2 h (B). Mitochondria staining was performed with MitoTracker deep red (red channel), and nuclear staining was performed using green nuclear dye (blue fluorescence). Negative control is displayed in row (C). Scale bar: 20 μm.

**Figure 3:**
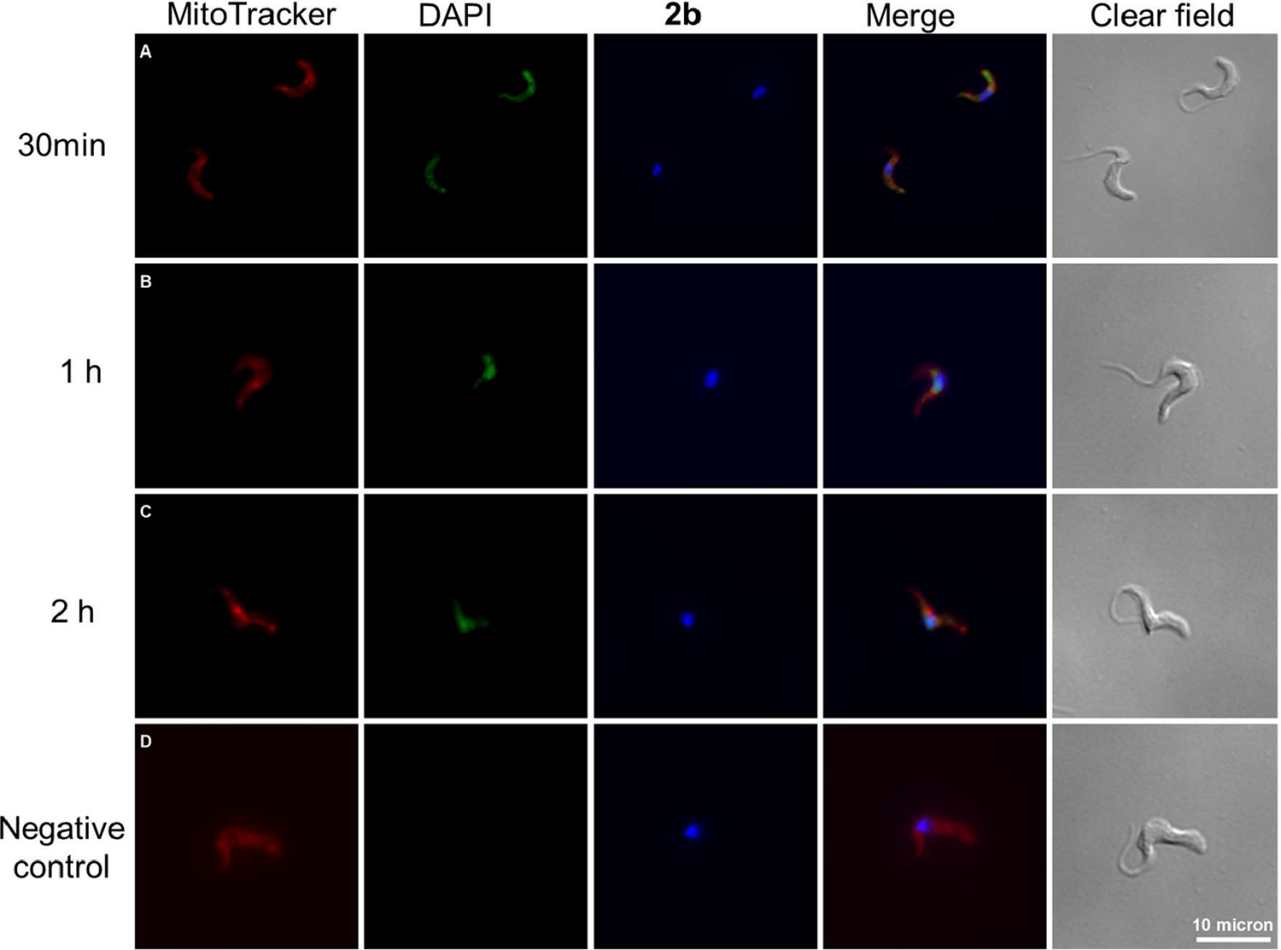
Fluorescence microscopy of *T. brucei* parasites treated with compound **2b**. Parasites were incubated with 5 μM compound **2b** (green channel) for 30 min (A); 1 h (B); and 2 h (C). Mitochondria staining was performed with MitoTracker deep red (red channel) and nuclear staining was performed using Green nuclear dye (blue fluorescence). Negative control is displayed in row (D). Scale bar: 10 μm.

### In Silico ADMET Properties

Given its strong therapeutic activity and in line with our focus on the benzothiazole scaffold as a pharmacophore to improve the drug-like nature of G4 ligands, we calculated the ADMET (absorption, distribution, metabolism, excretion/toxicity) profile of compound **2b** and compared the results with other commercial G4 ligands, including the porphyrin TMPyP4 and quarfloxin, a fluoroquinolone-based G4 ligand that progressed to Phase II clinical trials for neuroendocrine tumors (ClinicalTrials.gov identifier: NCT00780663),^52^ using the ADMET lab2.0 online server^53^ (Table 4).

**Table 4:** ADMET Properties of Compound 2b and G4 Ligands TMPyP4 and Quarfloxin^a^

	properties	compound 2b	TMPyP4	quarfloxin
physicochemical	MW	422.03	1362.37	604.26
	log*P*	4.625	4.448	5.034
	TPSA (Å^2^)	7.12	301.68	92.59
	nHA	2	20	9
	nHD	0	2	1
	nRot	3	8	7
medicinal chemistry	Lipinski	accepted	rejected	rejected
	SAscore	2.622	6.775	4.003
absorption	Caco-2 permeability (logcm/s)	–4.616 (yes)	–6.024 (no)	–5.068 (yes)
	MDCK permeability(cm/s)	1.9 × 10^–5^ (yes)	1.2 × 10^–5^ (yes)	1.6 × 10^–5^ (yes)
distribution	VD(L/kg)	3.16	–0.232	2.44
	BBB penetration*	0.511 (medium)	1 (high)	0.047 (low)
metabolism	CYP1A2 inhibitor/substrate*	0.946/0.936	0.102/0.943	0.149/0.79
	CYP2C19 inhibitor/substrate*	0.729/0.837	0.051/0.057	0.546/0.752
	CYP2C9 inhibitor/substrate*	0.199/0.669	0.024/0.007	0.612/0.545
	CYP2D6 inhibitor/substrate*	0.864/0.883	0.009/0.84	0.888/0.876
	CYP3A4 inhibitor/substrate*	0.602/0.384	0.004/0.249	0.927/0.92
excretion	CL(mL/min/kg)	6.93 (medium)	0.831 (low)	2.026 (low)
	*T*_1/2_**	0.215	0.004	0.053
toxicity	hERG blockers*	0.056 (low)	0.941 (high)	0.963 (high)
	H-HT*	0.021 (low)	0.988 (high)	0.96 (high)
	AMES toxicity*	0.983 (high)	0.048 (low)	0.813 (high)
	rat acute oral toxicity*	0.029 (low)	0.179 (low)	0.354 (medium)

a MW (molecular weight); log*P* (predicted octanol/water partition coefficient): optimal 0–3; nHA (number of hydrogen acceptors): optimal 0–12; *n*HD (number of hydrogen donors): optimal 0–7; *n*Rot (number of rotatable bonds): optimal 0–11; TPSA (topological polar surface area): optimal 0–140; Lipinski’s rule: MW ≤ 500, log*P* ≤ 5, *n*HA ≤ 10, *n*HD ≤ 5; SAscore (Synthetic accessibility score): ≥6 difficult to synthesize, <6 easy to synthesize; Caco-2 permeability: optimal > −5.15; MDCK permeability (cm/s): optimal >2 × 10^–6^ cm/s; VD (volume distribution): optimal 0.04–20 L/kg; CL (clearance): >15 “high clearance”, 5–15 “moderate clearance”, <5 “low clearance”; *T*_1/2_ (half-life); H-HT (human hepatotoxicity); *Probability of being positive (0–1); ** Probability of long *T*_1/2_.

The analysis revealed compound **2b**’s Lipinski’s rule compliance, suggesting favorable oral bioavailability as well as a low synthetic accessibility score. In contrast, TMPyP4 and quarfloxin violated Lipinski’s criteria and presented greater synthetic complexity. Compound **2b** and quarfloxin exhibited adequate permeability and optimal blood distribution, whereas TMPyP4 showed reduced oral absorption and distribution profiles. The BBB permeability predictions, particularly relevant for treating stage 2 of the sleeping sickness when the central nervous system (CNS) has been compromised by the parasite, revealed compound **2b**’s higher BBB penetration compared to quarfloxin, while TMPyP4 exhibited the highest BBB permeability among the three. In fact, structurally related benzothiazolium derivatives have already been reported to efficiently cross the BBB in vivo, thereby further supporting our in silico results.^54^ Compound **2b**, as well as quarfloxin, exhibited a significant likelihood of interaction with CYP450 isozymes, while TMPyP4 showed a lower overall interaction probability. Moreover, compound **2b** showed higher clearance and a longer half-life than TMPyP4 and quarfloxin, indicating sustained therapeutic effects with a potentially reduced dosing frequency. Finally, toxicity assessments suggested lower hERG inhibition and hepatotoxicity for **2b** than those for TMPyP4 and quarfloxin. However, the AMES indicated a higher mutagenicity risk for **2b** and quarfloxin relative to TMPyP4. Acute toxicity was predicted to be low for **2b** and TMPyP4 and to be high for quarfloxin.

Overall, compound **2b** exhibits favorable ADMET calculated properties, particularly in terms of oral bioavailability and systemic distribution, including adequate BBB penetration, supporting its potential as a candidate for further development. To strengthen the reliability of the ADMET predictions, additional computational tools were employed alongside the primary software, namely, pkCSM and SwissADME (Table S2–S3). The comparative analysis revealed a high degree of consistency among the results, thereby supporting the reliability of the in silico predicted properties.

### Binding to DNA G4

After evaluating the therapeutic activity of the 2-ethynyl benzothiazolium derivatives and identifying compounds **2b**, **2c**, **2d**, **2g**, and **2h** as promising antiparasitic agents, we investigated whether the in vitro antiparasitic activity of these derivatives could be associated with their G4 binding capability. This interest was driven by their structural similarity to ThT, a well-established G4-fluorescence probe. TMPyP4 was used as a reference G4 ligand.

The stabilization of G4 structures was initially evaluated by using the Förster resonance energy transfer (FRET) melting assay. This analysis evaluated the binding of the compounds against four G4-forming oligonucleotides and a hairpin duplex sequence to determine both G4:duplex and G4:G4 selectivity. The sequences used in this study (Table S4) included the human telomeric G4, found in *T. brucei* and several mammalian species,^55^ in potassium buffer (F21T K^+^, mixed parallel/hybrid G4) and sodium buffer (F21T Na^+^, antiparallel G4), a polymorphic G4 found in *T. brucei* ^19^ (FEbr1T, mixed G4 topology), the c-Myc promoter G4 (FmycT, parallel G4), a human mitochondrial G4 (Fmt6363T, hybrid G4), and a hairpin duplex DNA sequence (F10T). These G4 sequences were selected to represent a possible wide variety of G4 topologies present in the genome of *T. brucei*. Moreover, a duplex DNA sequence was also selected in order to study the potential selective binding of the compounds to either G4 or duplex DNA. The results are summarized in Figure 4 and Table S4.

**Figure 4:**
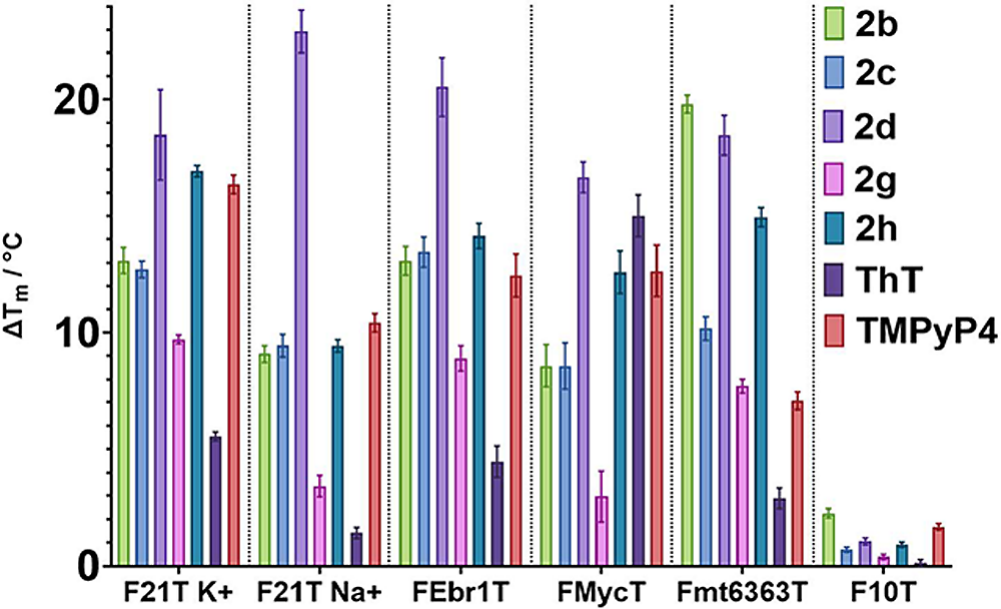
FRET melting assays for compounds **2b**, **2c**, **2d**, **2g**, and **2h**, ThT at 10 μM and TMPyP4 (1 μM as control) with different G-quadruplexes (F21T, FEbr1T, FMycT, and Fmt6363T) and duplex sequence (F10T).

The first general trend that can be observed is that all the 2-ethynyl benzothiazolium derivatives examined bind across all the G4s screened, except for **2g**, which was more selective and did not show binding to F21T Na^+^ and FMycT G4s. Moreover, all the compounds were better G4 binders than control ThT, except when binding the parallel G4 FMycT, where ThT showed similar binding to **2d** and **2h**. Among the 2-ethynyl benzothiazolium derivatives, compound **2d** showed the highest stability in the series for all G4s. **2d** turns out to be the largest and most hydrophobic among them. At the same time, all benzothiazolium analogues demonstrated selectivity for G4 structures over the duplex DNA F10T.

In more detail, for the G4 structure F21T in potassium buffer (F21T K^+^), the results indicated that most compounds exhibited significant stabilization (Δ*T*_m_ > 10 °C), with compounds **2d** and **2h** showing the highest stabilization values (Δ*T*_m_ = 18.5 and 16.9 °C, respectively). In sodium buffer (F21T Na^+^), compound **2d** demonstrated the highest G4 stabilization of all of the other tested analogues. A similar trend was observed with the unique *T. brucei* G4 sequence (FEbr1T), where compound **2d** exhibited the highest stabilization value (Δ*T*_m_ = 20.5 °C), while compounds **2b**, **2c**, and **2h** (Δ*T*_m_ = 13.1, 13.5, and 14.1 °C, respectively) achieved moderate stabilization levels. The induced stabilization of G4 FMycT followed a very similar pattern. However, ThT showed better binding in this sequence compared to other G4 structures, with Δ*T*_m_ = 15.0 °C vs Δ*T*_m_ = 1.4–5.5 °C for the other examined G4s. Lastly, for mitochondrial G4 (Fmt6363T), compounds **2b**, **2d**, and **2h** exhibited the highest stabilization. Notably, compound **2b** showed a marked preference for stabilizing the mitochondrial G4, with a Δ*T*_m_ of 19.8 °C compared to 8.5–13.1 °C for other G4 sequences. Additionally, FRET analysis was conducted for compound **2b** with the mt6363 sequence under increasing concentrations of potassium buffer (Figure S1). The results indicated that the level of G4 stabilization by compound **2b** increased with higher potassium concentrations.

To further investigate the DNA stabilization potential and effects on the topology of ligand **2b** on G4s and duplex structures, circular dichroism (CD) spectroscopy was employed on four distinct oligonucleotide sequences: telo23, mt6363, Ebr1, and ds26 (Figures 5 and S2, S3 and Table S6). Quantitative changes in the secondary and tertiary structures of the G4 motifs upon increasing concentrations of compound **2b** were assessed using principal component analysis (PCA) and singular value decomposition (SVD) of the CD data.^56^ The results indicated that the incremental addition of **2b** induces subtle alterations in the tertiary topology of the analyzed G4 structures. Specifically, while the hybrid topology of telo23 is maintained, higher concentrations of **2b** promote an increase in the proportion of antiparallel G4 structures (Figure 5). For the mt6363 and Ebr1 G4s, ligand binding shifts the topology toward the parallel form, diminishing the prevalence of the hybrid topology observed in the absence of the compound (Figure S2). In contrast, the effect of **2b** on the secondary parameters of G4s was less pronounced.

**Figure 5:**
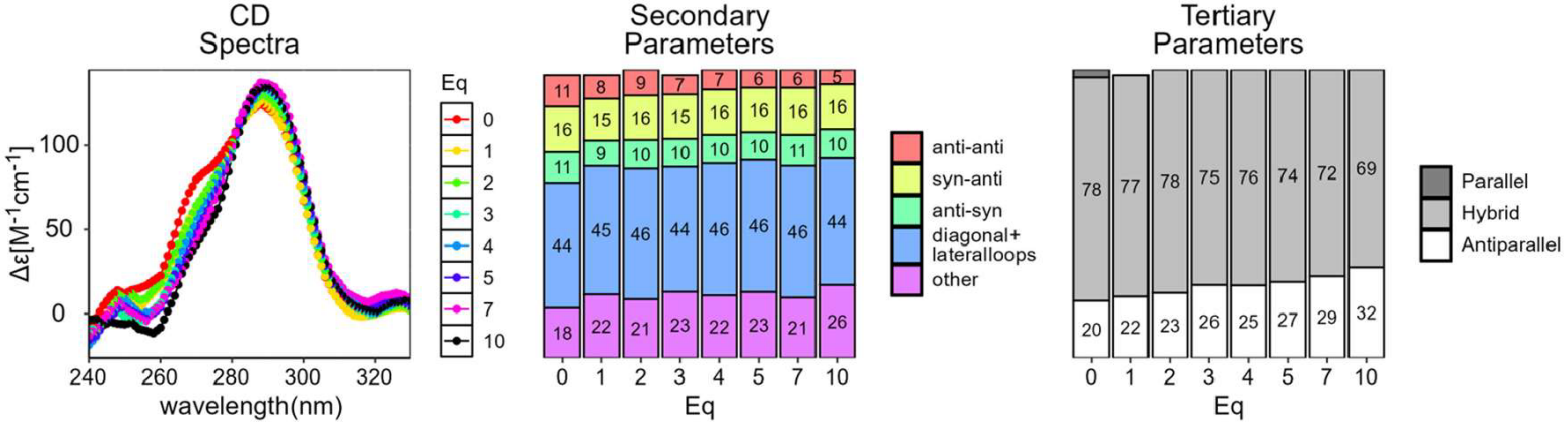
Left, CD spectra of telo23 with different equivalents of compound 2b (vertical color scale on the side). Buffer = 100 mM potassium phosphate, pH 7.4. Oligonucleotide concentration = 5 μM; center and right, SVD analysis of the influence of the number of equivalents of **2b** on the secondary and tertiary parameters.

Ultraviolet–visible (UV–vis) absorbance spectroscopy titrations were carried out to quantitatively assess the binding affinity of compounds **2b** and **2c** to G4 sequences (telo23, ebr1, and mt6363) and the duplex sequence (ds26) (Figures 6 and S4). Consistent with the FRET analysis, compound **2b** exhibited a higher affinity for the mt6363 G4 structure compared with the other G4 sequences. Furthermore, the analysis confirmed its selectivity for G4 structures over duplex DNA.

**Figure 6:**
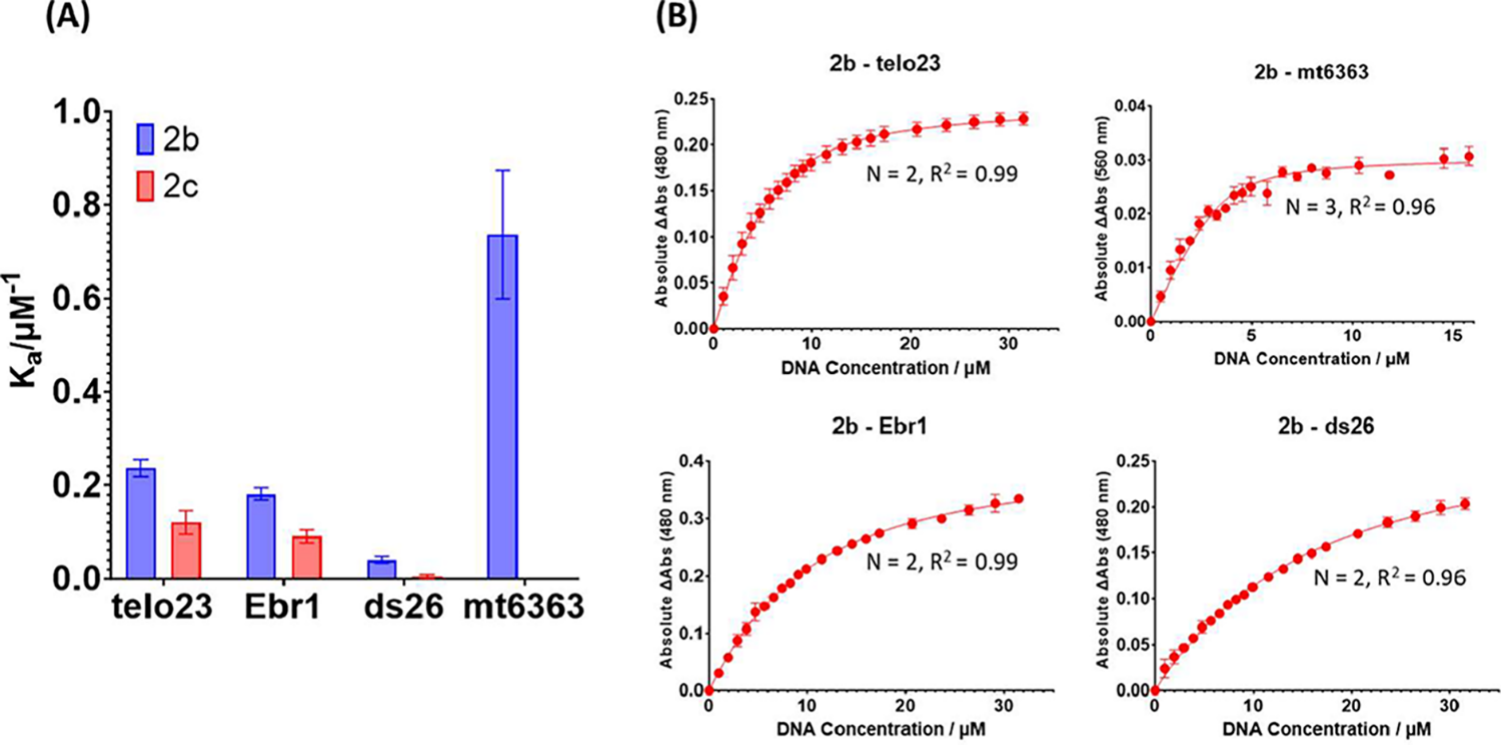
(A) Equilibrium binding constants calculated for compounds **2b** and **2c** with several G-quadruplexes (telo23, Ebr1, and mt6363) and a duplex DNA (ds26) sequence. (B) UV–vis titration of 2b with telo23, mt6363, Ebr1, and ds26. Buffer = 100 mM potassium phosphate, pH 7.4. Ligand concentration: 10 μM.

## Conclusions

In this research, we explored the structural extension of the benzothiazole scaffold in the ThT probe by introducing various 2-ethenyl aromatic and heteroaromatic moieties. Our findings demonstrated that these C-2 modifications improved both the G4 binding affinity and the biological activity of the resulting benzothiazolium derivatives. Specifically, we observed that the derivative functionalized with the (*N,N*-dimethylamino)­phenyl moiety, compound **2b**, exhibited remarkable activity against bloodstream forms of *T. brucei* (IC_50_ = 0.019 nM), 46,000-fold more active than the reference drug fexinidazole, along with an exceptional selectivity index of 79,206. To the best of our knowledge, this represents one of the most potent antitrypanosomal agents reported, with an outstanding therapeutic window. Furthermore, a comparison with ThT highlighted the critical role of the ethenyl bridge and the absence of C-6 substitution in significantly enhancing antitrypanosomal activity. For antileishmanial activity, although the julolidine-substituted derivative **2h** exhibited the highest potency against *L. major* promastigotes, the triphenylamine-substituted **2c** demonstrated greater therapeutic potential due to its selectivity index (SI = 252) and greater antileishmanial activity than miltefosine and AmB. In the *L. major* amastigotes assays, compound **2b** exhibited outstanding potency and selectivity, with an IC_50_ of 0.48 nM and an SI of 46,151, markedly outperforming **2c** as well as the standard antileishmanial drugs miltefosine and AmB. Furthermore, the predicted ADMET properties suggest that compound **2b** has a more favorable drug-like profile than the reference G4 ligands, reinforcing its potential as a promising candidate for further development.

G4 binding studies using FRET-melting, CD titrations, and UV–vis titrations showed that the most active benzothiazolium derivatives also exhibit significant G4 stabilization and selectivity over duplex DNA. Among them, compound **2d** displayed the highest stabilization across all tested G4 motifs, which may be attributed to its larger size and increased hydrophobicity. In contrast, ThT showed comparatively lower stabilization of these secondary structures. Fluorescence microscopy studies on compound **2b** confirmed its intracellular localization mainly within the nucleus and kinetoplast, supporting the hypothesis that G4 structures within the parasite genome could serve as potential molecular and therapeutic targets. Although the most active derivatives exhibit strong DNA-G4 binding, other factors such as membrane permeability, access to the nucleus, and access to G-quadruplexes when formed within the nucleus (and/or alternative mechanisms of action such as interactions with RNA-G4s), may also affect the compounds’ activity, toxicity, and efficacy as potential antiparasitic treatments. Overall, these results highlight the potential of benzothiazolium derivatives as G4-targeting ligands and antiparasitic agents, while emphasizing the need for further investigations into their cellular uptake and broader biological effects to fully elucidate their mechanism of action.

## Methods

### Antiparasitic Activity

The antitrypanosomal activity of the compounds against the bloodstream form of *T. brucei* was evaluated using the alamarBlue assay (ThermoFisher scientific).^19,57^ The stock solutions of the compounds were prepared in DMSO, with the final DMSO percentage in each well maintained below 1%. 2 × 10^4^ parasites per mL were incubated at 37 °C, 5% CO_2_ in 96-wells plates (50 μL/well) either alone or in the presence of increasing concentrations of compounds for 72 h. After the incubation period, 20 μL of resazurin solution (110 ng/mL) was added to each well, and the parasites were further incubated for 4 h at 37 °C. Subsequently, cell lysis was performed using 50 μL per well of 3% SDS. The plate was then incubated at 37 °C for an additional hour, followed by measurement of fluorescence intensity using an Infinite F200 plate reader (Tecan Austria, GmbH). The excitation wavelength was set at 550 nm, and emission was recorded at 590 nm. The results are expressed as the concentration of the compound that reduces cell growth by 50% compared to untreated control cells (IC_50_). Data were presented as the average of at least three independent measurements, each conducted under triplicate conditions.

The antileishmanial activity of the compounds against the promastigote form of *L. major*, the clinically relevant form was determined using an MTT-based assay (Sigma-Aldrich).^58^ Stock solutions of the compounds were prepared in DMSO with the final DMSO percentage in each well maintained below 1%. 4 × 10^6^ parasites per mL were incubated at 28 °C in 96-well plates (50 μL/well) either alone or in the presence of increasing concentrations of compounds DMSO for 72 h. After the incubation period, 10 μL of MTT (5 mg/mL) was added to each well, and the parasites were incubated for 4 h at 28 °C. Subsequently, cell lysis was performed using 50 μL/well of 20% SDS. The plate was then incubated at 37 °C for an additional hour, and then absorbance was measured at a wavelength of 540 using an Infinite F200 plate reader (TECAN Austria, GmbH). The IC_50_ was calculated as described above. Data were presented as the average of at least three independent measurements, each conducted under triplicate conditions.

The antileishmanial activity of the compounds against the intracellular form of *L. major* was evaluated using the Luciferase Assay System kit from Promega. THP-1 cells were seeded at a density of 3 × 10^5^ cells/mL in 96-well plates (100 μL/well) and treated with 20 ng/mL PMA (phorbol 12-myristate 13-acetate, Sigma) for 48 h to induce differentiation. Postdifferentiation, the cells were washed, and fresh medium was added (100 μL/well) before being reincubated. Following an additional 24 h, the wells were washed, and 50 μL of *L. major* parasites at a density of 1.5 × 10^6^ parasites/mL (suspended in RPMI-1640 medium 5% hiFBS and 5% penicillin/streptomycin) were added to the wells. The plates were then incubated at 35 °C in 5% CO_2_. After 24 h, the wells were washed three times with PBS, and fresh medium with increasing concentrations of compounds was added (100 μL/well). After another 72 h of incubation, the cells were then treated with 25 μL of lysis buffer and stored at −80 °C. Following 24 h, 25 μL of luciferase enzyme substrate was added to each well, and the luciferase activity of the amastigotes was determined using a TECAN Infinite F200 microplate reader. Results are reported as the concentration of compounds that reduce parasite growth by 50% compared to untreated control-infected macrophages (IC_50_). Data were presented as the average of at least three independent measurements, each conducted under triplicate conditions.

Controls for the AlamarBlue, MTT, and Cell Tilter assays were run at the different compound concentrations to assess optical interference. Detectable background (fluorescence and absorbance) was limited to some compounds (mainly, **2b** and **2h**) at 100 μM and was negligible at ≤1 μM (all IC_50_ determinations).

### Cell Titer Glo Luminescent Cell Viability Assay

The trypanocidal activity of the more active compounds was also assessed by the Cell Titer Glo assay (Promega). To do so, 50 BSF *T. brucei* parasites per well were incubated in 96-wells plates in the presence of increasing concentrations of compounds for 72 h at 37 °C. Ten μL portion of Cell Titer Glo was then added to every well, and after 10 min of incubation at room temperature, the luminescence of the plates was recorded using the Infinite F200 plate reader (TECAN Austria, GmbH). The results herein are expressed as the compound concentration that reduces cell growth by half versus untreated control cells (IC_50_) using SigmaPlot (Four Parameter Logistic Curve). Presented data are the mean value of three independent measurements, all of them conducted under triplicate conditions.

### Cytotoxicity

The cytotoxic effect of the compounds was evaluated in MRC-5, THP-1, HeLa, and HT-29 cell lines, through the alamarBlue assay (ThermoFisher scientific).^19,57^ Stock solutions of the compounds were prepared in DMSO, with the final DMSO concentration in each well maintained below 1%. Prior to compound addition, 5 × 10^3^ cells per mL (MRC-5, HeLa or HT-29 cells) were seeded in 96-well plates (100 μL/well) and incubated for 24 h at 37 °C, 5% CO_2_ to allow cell attachment. Subsequently, compounds were added at various concentrations ranging from 0 to 100 μM, and the plates were incubated for 72 h. For THP-1, cells were seeded at a density of 3 × 10^5^ cells/mL in 96-well plates (100 μL/well) and treated with 20 ng/mL PMA (phorbol 12-myristate 13-acetate, Sigma) for 48 h. Postdifferentiation, the cells were washed, and fresh medium was added (100 μL/well) before being reincubated. Following another 48 h, the wells were washed again, and fresh medium with increasing concentrations of compounds was added (100 μL/well). After the incubation period of 72 h, 20 μL of resazurin solution (110 ng/mL) was added to each well, and the cells (MRC-5, THP-1, HeLa, and HT-29) were incubated for an additional 4 h at 37 °C. Subsequently, cells were lysed with 50 μL of 3% (for MRC-5, HeLa, and HT-29 cells) or 20% (for THP-1 cells) SDS, which was added to each well. The plates were incubated at 37 °C for an additional hour, and subsequently, the fluorescence intensity was measured using the Infinite F200 plate reader (TECAN Austria, GmbH), exciting at 550 nm and recording the emission at 590 nm. The obtained results were expressed as the concentration of compound that reduces cell growth by 50% compared to untreated control cells (IC_50_). Data are presented as the average of at least three independent measurements, each conducted under triplicate conditions.

## Supplementary Material



## Notes

Synthesis and characterization of benzothiazolium derivatives, parasite and cell culturing, fluorescence microscopy, In silico ADMET properties, FRET-melting assays, CD titrations, UV–vis titrations, pharmacokinetic and toxicity (ADMET) properties, G4-forming and duplex sequences, CD spectra, and HPLC chromatograms (PDF)

## α

Nanoscopy-UGR Lab, Departamento de Fisicoquímica, Facultad de Farmacia, Universidad de Granada, Calle Prof. Vicente Callao, Beiro, 18011 Granada, Spain
