# Supplementary material for: Extending Thioflavin T Fluorescence Probe to 2‑Ethenyl-benzothiazole Derivatives: Drug-like Quadruplex Ligands with Potent Antitrypanosomatid Activity
Source: ACS Infect Dis. 2025 Nov 4;11(11):3344–54. doi: 10.1021/acsinfecdis.5c00691 (PMC12643529; doi:10.1021/acsinfecdis.5c00691)
Supplement: Supplementary file 1 [file id5c00691_si_001.pdf]

Supporting Information

Extending thioflavin T fluorescence probe to 2-ethenyl-benzothiazole derivatives: drug-like quadruplex ligands with potent anti-trypanosomatid activity

Raquel C. R. Gonçalves[1,2], Pablo Peñalver[3,4], Nina M. Allen[5], Efres Belmonte-Reche[6,7,8], Belén García-Pérez[3], Susana P. G. Costa[1], Y. Jennifer Jiang[5], José María Pérez-Victoria[3], M. Carmen Galan[5],* M. Manuela M. Raposo[1],* Juan Carlos Morales[3]*

[1] Centre of Chemistry, University of Minho, Campus of Gualtar, 4710-057 Braga, Portugal.
[2] Advanced (Magnetic) Theranostic Nanostructures Lab, International Iberian Nanotechnology Laboratory, 4715-330 Braga, Portugal.
[3] Departamento de Bioquímica y Farmacología Molecular, Instituto de Parasitología y Biomedicina López Neyra, CSIC, PTS Granada, Avenida del Conocimiento, 17, Armilla, 18016 Granada, Spain.
[4] Nanoscopy-UGR Lab, Departamento de Fisicoquímica, Facultad de Farmacia, Universidad de Granada, Calle Prof. Vicente Callao, Beiro, 18011 Granada, Spain.
[5] School of Chemistry, University of Bristol, Bristol BS8 1TS, United Kingdom.
[6] GENYO, Centre for Genomics and Oncological Research: Pfizer/University of Granada / Andalusian Regional Government, PTS Granada, Av. de la Ilustración, 114, 18016 Granada, Spain.
[7] Department of Biochemistry and Molecular Biology II, Faculty of Pharmacy, University of Granada, Granada, Spain.
[8] Instituto de Investigación Biosanitaria ibs.GRANADA, Hospital Virgen de las Nieves, Granada, Spain.

*Corresponding authors:
M.C.Galan@bristol.ac.uk
mfox@quimica.uminho.pt
jcmorales@ipb.csic.es

Table of contents

1. Materials and methods S2

1.1. Chemistry S2

Synthesis and characterization of benzothiazolium derivatives **2a-j** S3

1.2. Biological methods S7

Parasite and cell culturing S7

Fluorescence microscopy S7

In silico ADMET properties S8

1.3. Biophysical methods S8

FRET-melting assays S8

CD titrations S9

UV-vis titrations S10

Table S1. Synthesis and photophysical data in acetonitrile solution (absorption, λ_{abs}, fluorescence emission maxima, λ_{fluo}, molar absorptivity, $\mathcal{E}$ and fluorescence quantum yield, ϕ_F) for the 2-ethenyl-benzothiazolium derivatives **2a-j**. S10

Table S2. Pharmacokinetic and toxicity (ADMET) properties of the compound **2b** and G4 ligands TMPyP4 and quarfloxin, according to pkCSM software.[10] S12

Table S3. Pharmacokinetic (ADME) properties of the compound **2b** and G4 ligand Quarfloxin, according to SwissADME software.[11] S13

Table S4. G4-forming and duplex sequences included in the FRET-melting assays. S13

Table S5. Results of the FRET-melting assay performed on derivatives **2b**, **2c**, **2d**, **2g** and **2h**. S14

Figure S1. FRET melting analysis for compound **2b** (10 μM), ThT (10 μM) and TMPyP4 (1 μM) with mt6363 in increasing concentrations of K^+. S14

Table S6. G4-forming and duplex sequences included in the CD analysis. S15

Figure S2. CD spectra of (A) mt6363 with different equivalents of compound **2b** and analysis of the influence of the number of equivalents of **2b** on the secondary and tertiary parameters; (B) Ebr1 with different equivalents of compound **2b** and analysis of the influence of the number of equivalents of **2b** on the secondary and tertiary parameters. Buffer = 100 mM potassium phosphate, pH 7.4. Oligonucleotide concentration = 5 μM. S15

Figure S3. CD spectra of ds26 with different equivalents of compound **2b**. Buffer = 100 mM potassium phosphate, pH 7.4. Ligand concentration = 10 μM. S16

Figure S4. UV-vis analysis of compound **2c** with (A) telo23, (B) Ebr1 and (C) ds26 sequences. S16

Figure S5. HPLC chromatogram of compound **2a**. S17

Figure S6. HPLC chromatogram of compound **2b**. S17

Figure S7. HPLC chromatogram of compound **2c**. S18

Figure S8. HPLC chromatogram of compound **2d**. S18

Figure S9. HPLC chromatogram of compound **2e**. S19

Figure S10. HPLC chromatogram of compound **2f**. S19

Figure S11. HPLC chromatogram of compound **2h**. S20

Figure S12. HPLC chromatogram of compound **2i**. S20

Figure S13. HPLC chromatogram of compound **2j**. S21

References S21

1. Materials and methods

1.1. Chemistry

NMR spectra were obtained on a Bruker Avance III 400 at an operating frequency of 400 MHz for ^{1}H and 100.6 MHz for ^{13}C, using the solvent peak as an internal reference (δ relative to TMS). Peak assignments were supported by spin decoupling-double resonance and bidimensional heteronuclear techniques. High-resolution mass spectra (HRMS) were obtained on an ESI/quadrupole mass spectrometer (WATERS, ACQUITY H CLASS). All reagents were

purchased from Sigma-Aldrich, Acros, and Fluka and used as received. Thin-layer chromatography (TLC) was carried out on 0.25 mm thick precoated silica plates (Merck Fertigplatten Kieselgel $60F_{254}$), and spots were visualized under ultraviolet (UV) light. Fluorescence spectra were collected using a FluoroMax-4 spectrofluorometer. UV-visible absorption spectra (200–700 nm) were obtained using a Shimadzu UV/2501PC spectrophotometer. Fluorescence quantum yields were determined using Nile Red in dioxane and Nile Blue in methanol as standards ($\phi_F = 0.7$ and $\phi_F = 0.3$, respectively).[1] Purity of the synthesized compounds was confirmed by HPLC and all compounds showed > 95 % purity. The synthesis of benzothiazolium derivatives **2a**, **2b**, **2c**, **2e**, **2g**, and **2h** have been described previously by our research group and others[2–4].

Synthesis and characterization of benzothiazolium derivatives 2a-j

A solution of aromatic aldehydes **1a-1j** (1 mmol), 2,3-dimethylbenzothiazolium iodide (1 mmol), and a catalytic amount of piperidine in ethanol (10 mL) was heated at reflux for 6 h. The reaction mixture was allowed to cool to room temperature and the final compounds were purified by recrystallization from dichloromethane, diethyl ether and washed with cold ethanol.

*(E)-3-Methyl-2-styrylbenzo[d]thiazol-3-ium iodide (**2a**)*

^{1}H NMR (400 MHz, DMSO-d_6) δ = 4.37 (s, 3H, N^+CH_3), 7.55–7.58 (m, 3H, H-3′, H-4′- and H-5′), 7.81 (dt, 1H, J = 1.2 and 7.4 Hz, H-6), 7.89 (dt, 1H, J = 1.2 and 7.4 Hz, H-5), 8.05–8.07 (m, 2H, H-2′ and H-6′), 8.06 (d, 1H, J = 16.0 Hz, H-b) 8.22 (d, 1H, J = 16.0 Hz, Ha), 8.25–8.28 (m, 1H, H-7), 8.44 (dd, 1H, J = 1.2 and 7.6 Hz, H-4) ppm. MS (ESI) *m/z* 252 $[M]^+$, 230, 207, 102.

*(E)-2-(4-(Dimethylamino)styryl)-3-methylbenzo[d]thiazol-3-ium iodide (**2b**)*

^{1}H NMR (400 MHz, DMSO-d_6) δ = 3.10 (s, 6H, $N(CH_3)_2$), 4.22 (s, 3H, N^+CH_3), 6.63 (d, 2H, J = 8.8 Hz, H-3′and H-5′), 7.62 (d, 1H, J = 15.4 Hz, H-b), 7.67 (dt, 1H, J = 0.9 and 7.8 Hz, H-6), 7.78 (dt, 1H, J = 0.9 and 7.8 Hz, H-5), 7.90 (d, 2H, J = 8.8 Hz, H-2′ and H-6′), 8.06 (d, 1H, J = 15.4 Hz, H-a), 8.07–8.09 (m, 1H, H-7), 8.28 (dd, 1H, J = 0.9 and 8.0 Hz, H-4) ppm. MS (ESI) *m/z* 297, 295 $[M]^+$, 148.

*(E)-2-(4-(Diphenylamino)styryl)-3-methylbenzo[d]thiazol-3-ium iodide (**2c**)*

^{1}H NMR (400 MHz, DMSO-d_6): δ = 4.25 (s, 3H, CH_3), 6.88 (d, J = 8.8 Hz, 2H, H-2' and H-6'), 7.17-7.24 (m, 6H, 2 x (H-2", H-4" and H-6")), 7.70-7.44 (m, 4H, 2 x (H-3" and H-5")), 7.71 (dd, J = 0.8 and 8.0 Hz, 1H, H-6), 7.76 (d, J = 16 Hz, 1H, H-b), 7.81 (dt, J = 1.2 and 8.4 Hz, 1H, H-5), 7.90 (d, J = 8.8 Hz, 2H, H-3' and H-5'), 8.09 (d, J = 16 Hz, 1H, H-a), 8.17 (d, J = 8.4 Hz, 1H, H-7), 8.36 (dd, J = 0.8 and 8.0 Hz, 1H, H-4) ppm. MS (ESI) *m/z* 419 $[M]^+$, 297, 223, 177, 126, 102.

*(E)-2-(2-(5-(4'-(Diphenylamino)-[1,1'-biphenyl]-4-yl)furan-2-yl)vinyl)-3methylbenzo[d]thiazol-3-ium iodide (**2d**)*

Red solid (η = 30 %). ^{1}H NMR (DMSO-d_6): δ = 4.35 (s, 3H, N^+CH_3), 7.04-7.11 (m, 8H, H-2"', H-6"' 2x (H-2"", H-4"" and H-6"")), 7.32-7.36 (m, 2H, 2x (H-3"" and H-5"")), 7.41 (d, J = 3.6 Hz, 1H, H-3'), 7.49 (d, J = 4.0 Hz, 1H, H-4'), 7.68-7.72 (m, 3H, H-3"', H-5"' and H-b), 7.76 (dt, J = 1.2 and 8.4 Hz, 1H, H-6), 7.80 (d, J = 8.8 Hz, 2H, H-3" and H-5"), 7.85 (dt, J = 1.2 and 8.4 Hz, 1H, H-5), 8.08-8.12 (m, 3H, H-a, H-2" and H-6"), 8.22 (d, J = 8.0 Hz, 1H, H-4), 8.40 (d, J = 7.6 Hz, 1H, H-7) ppm. ^{13}C NMR (DMSO-d_6): δ = 36.15 (CH_3), 109.40 (C-b), 110.65 (C-3'), 116.57 (C-4), 122.89 (C-2"' and C-6"'), 123.48 (C-4""), 124.15 (C-4'), 124.24 (C-7), 124.35 (C-2"" and C-6""), 125.66 (C-2" and C-6"), 126.59 (C-3" and C-5"), 127.01 (C-1"), 127.55 (C-7a), 127.65 (C-3"' and C-5"'), 128.15 (C-6), 129.29 (C-5), 129.64 (C-3"" and C-5""), 132.55 (C-4"'), 133.11 (C-a), 140.52 (C-4"), 142.05 (C-3a), 146.85 (C-1""), 147.27 (C-1"'), 150.32 (C-5'), 158.36 (C-2'), 170.83 (C-2) ppm. HRMS (ESI) *m/z*: $[M\text{-}I^-]^+$ calcd for $C_{38}H_{29}N_2OS^+$ 561.2001, found 561.2004.

*(E)-3-Methyl-2-(2-(1-methyl-1H-pyrrol-2-yl)vinyl)benzo[d]thiazol-3-ium iodide (**2e**)*

^{1}H NMR (DMSO-d_6): δ = 3.89 (s, 3H, CH_3), 4.21 (s, 3H, N^+CH_3), 6.37–6.39 (m, 1H, H-4′), 7.37–7.38 (m, 1H, H-3′), 7.45 (dd, 1H, J = 1.5 and 4.2 Hz, H-5′) 7.51 (d, 1H, J = 15.2 Hz, H-b), 7.67 (dt, 1H, J = 1.1 and 7.7 Hz, H-6), 7.78 (dt, 1H, J = 1.1 and 7.7 Hz, H-5), 7.95 (d, 1H, J = 15.2 Hz, H-a), 8.08–8.11 (m, 1H, H-7), 8.30 (dd, 1H, J = 0.9 and 8.1 Hz, H-4) ppm. MS (ESI) *m/z* 257, 256, 255 $[M]^+$.

*(E)-3-Methyl-2-(2-(5-(1-methyl-1H-pyrrol-2-yl)thiophen-2-yl)vinyl)benzo[d]thiazol-3-ium iodide (**2f**)*

Dark brown solid (η = 20 %). ^{1}H NMR (DMSO-d_6) δ = 3.85 (s, 3H, NCH_3), 4.26 (s, 3H, N^+CH_3), 6.14-6.15 (m, 1H, H-4"), 6.60 (dd, 1H, J = 2.0 and 4.0 Hz, H-3"), 7.00 (dd, 1H, J = 2.0 and 4.0 Hz, H-5"), 7.40 (d, 1H, J = 4.2 Hz, H-4'), 7.52 (d, 1H, J = 15.4 Hz, H-b), 7.72 (dt, 1H, J = 1.0 and 8.2 Hz, H-6), 7.81 (dt, 1H, J = 1.0 and 8.2 Hz, H-5), 8.15-8.17 (m, 1H, H-7), 8.37-8.38 (m, 1H, H-4), 8.38 (d, 1H, J = 15.4 Hz, H-a). ^{13}C NMR (DMSO-d_6): δ = 35.8 (NCH_3), 36.0 (N^+CH_3), 108.7 (C-b), 109.8 (C-4), 112.1 (C-7), 116.2 (C-3"), 124.1 (C-3'), 124.7(C-7a), 125.0 (C-4"), 126.0 (C-6), 127.2 (C-5), 128.0 (C-5"), 129.0 (C-7), 136.0 (C-5'), 137.4 (C-a), 140.7 (C-3a), 141.7 (C-2'), 143.2 (C-2"), 170.7 (C-2). HRMS (ESI) *m/z*: $[M\text{-}I^-]^+$ calcd for $C_{19}H_{17}N_2S_2^+$ 337.0833, found 337.0833.

*(E)-2-(2-(4-(Dimethylamino)naphthalen-1-yl)vinyl)-3-methylbenzo[d]thiazol-3-ium iodide (**2g**)*

^{1}H NMR (400 MHz, DMSO-d_6): δ = 3.04 (s, 6H, $N(CH_3)_2$), 4.34 (s, 3H, N^+CH_3), 7.19 (d, 1H, J = 8.4 Hz, H-3′), 7.60 (dt, 1H, J = 1.3 and 7.0 Hz, H-6), 7.70 (dt, 1H, J = 1.3 and 7.0 Hz, H-5), 7.76 (dt. 1H, J = 1.2 and 8.0 Hz, H-6′), 7.85 (dt, 1H, J = 1.2 and 8.0 Hz, H-7′), 7.96 (d, 1H, J = 15.2 Hz, H-b) 8.18–8.22 (m, 2H, H-5′- and H-8′), 8.38 (dd, 1H, J = 0.4 and 8.0 Hz, H-7), 8.44 (dd, 1H, J = 8.4 Hz, H-2′), 8.49–8.51 (m, 1H, H-4), 8.81 (d, 1H, J = 15.2 Hz, H-a) ppm. MS (ESI) *m/z* 347, 346, 345 $[M]^+$, 223, 173, 166, 126.

*(E)-3-Methyl-2-(2-(2,3,6,7-tetrahydro-1H,5H-pyrido[3,2,1-ij]quinolin-9-yl)vinyl)benzo[d]thiazol-3-ium iodide (**2h**)*

^{1}H NMR (400 MHz, DMSO-d_6): δ = 1.88 (m, 4H, 2 x (CH_2)), 2.72 (t, 4H, J = 6.0 Hz, 2 x (CH_2)), 3.37 (m, 4H, 2 x (CH_2)), 4.14 (s, 3H, N^+CH_3), 7.42 (d, 1H, J = 14.8 Hz, H-b), 7.48 (s, 2H, H-2' and H-6'), 7.61 (dt, 1H, J = 1.2 and 7.2 Hz, H-6), 7.72 (dt, 1H, J = 1.2 and 7.2 Hz, H-5), 7.86 (d, J = 14.8 Hz, H-a), 8.00 (d, J = 8.4 Hz, H-7), 8.21 (dd, J = 0.8 and 8 Hz, H-4) ppm. MS (ESI) *m/z* 348 $[M+1]^+$, 347 $[M]^+$, 223, 174, 140.

*(E)-2-(2-(5-(4-(Diphenylamino)phenyl)thiophen-2-yl)vinyl)-3-methylbenzo[d]thiazol-3-ium iodide (**2i**)*

Dark violet solid (η = 8 %). ^{1}H NMR (400 MHz, DMSO-d_6): δ = 4.27 (s, 3H, CH_3), 6.98 (dd, *J* = 2.0 and 8.8 Hz, 2H, H-2" and H-6"), 7.09-7.16 (m, 6H, 2x (H-2"', H-4"' and H-6"')), 7.34-7.39 (m, 4H, 2x (H-3"' and H-5"')), 7.59 (d, *J* = 15.6 Hz, 1H, H-b), 7.64 (d, *J* = 4.0 Hz, 1H, H-3'), 7.68 (d, *J* = 9.2 Hz, 2H, H-3" and H-5"), 7.75 (dt, *J* = 0.8 and 7.2 Hz, 1H, H-6), 7.84 (dt, *J* = 1.2 and 8.4 Hz, 1H, H-5), 7.93 (d, *J* = 4.0 Hz, 1H, H-4'), 8.19 (d, *J* = 8.4 Hz, 1H, H-4), 8.37 (d, *J* = 9.2 Hz, 1H, H-7), 8.41 (d, *J* = 15.6 Hz, 1H, H-a) ppm. ^{13}C NMR (100.6 MHz, DMSO-d_6): δ = 36.07 (CH_3), 110.60 (C-b), 116.52 (C-4), 121.64 (C-2" and C-6"), 124.13 (C-7), 124.22 (C-4"'), 124.83 (C-3'), 125.07 (C-2"' and C-6"'), 125.57 (C-4"), 127.15 (C-3" and C-5"), 127.52 (C-7a), 128.10 (C-6), 129.25 (C-5), 129.81 (C-3"' and C-5"'), 137.26 (C-4'), 137.64 (C-3a), 140.97 (C-a), 141.98 (C-5'), 146.34 (C-1"'), 148.50 (C-1"), 151.67 (C-2'), 170.96 (C-2) ppm. HRMS (ESI) *m/z*: $[M-I^-]^+$ calcd for $C_{32}H_{25}N_2S_2^+$ 501.1454, found 501.1462.

*(E)-2-(2-(5'-(4-(Diphenylamino)phenyl)-[2,2'-bithiophen]-4-yl)vinyl)-3-methylbenzo[d]thiazol-3-ium iodide (**2j**)*

Dark violet solid (η = 15 %). ^{1}H NMR (400 MHz, DMSO-d_6): δ = 4.29 (s, 3H, CH_3), 6.97 (dd, *J* = 2.0 and 8.8 Hz, 2H, H-2"" and H-6""), 7.06-7.13 (m, 6H, 2x (H-2"", H-4"" and H-6"")), 7.32-7.36 (m, 4H, 2x (H-3"" and H-5"")), 7.48 (d, *J* = 4 Hz, 1H, H-3"), 7.57 (d, *J* = 4.0 Hz, 2H, H-4" and H-3'), 7.59 (d, *J* = 8.8 Hz, 2H, H-3"' and H-5"'), 7.61 (d, *J* = 15.6 Hz, 1H, H-b), 7.75 (dt, *J* = 0.8 and 8.0 Hz, 1H, H-6), 7.84 (dt, *J* = 1.2 and 7.2 Hz, 1H, H-5), 7.91 (d, *J* = 4.0 Hz, 1H, H-4'), 8.19 (d, *J* = 8.4 Hz, 1H, H-4), 8.38 (d, *J* = 8.8 Hz, 1H, H-7), 8.40 (d, *J* = 15.2 Hz, 1H, H-a) ppm. ^{13}C NMR (100.6 MHz, DMSO-d_6): δ = 36.12 (CH_3), 111.12 (C-b), 116.57 (C-4), 122.41 (C-2"' and C-6"'), 123.79 (C-4""), 124.14 (C-7), 124.40 (C-3"), 124.63 (C-2"" and C-6""), 125.67 (C-3'), 126.32 (C-4"') 126.61 (C-3"' and C-5"'), 127.60 (C-7a), 127.91 (C-4"), 128.17 (C-6), 129.30 (C-5), 129.72 (C-3"" and C-5""), 133.55 (C-5"), 137.39 (C-7), 137.55 (C-5'), 140.51 (C-a), 141.98 (C-3a), 144.24 (C-2'), 145.02 (C-2"), 146.61 (C-1""), 147.52 (C-1"'), 170.85 (C-2) ppm. HRMS (ESI) *m/z*: $[M-I^-]^+$ calcd for $C_{36}H_{27}N_2S_3^+$ 583.1331, found 583.1343.

1.2. Biological methods

Parasite and cell culturing

Trypanosoma brucei (Lister 427, antigenic type MiTat 1.2, clone 221a, bloodstream forms, “single marker” S427 (S16)) [5] were cultured at 37 °C, 5 % CO_2 in HMI-9 medium supplemented with 10 % heat-inactivated fetal bovine serum (hiFBS, Invitrogen), as previously described.[6]

Leishmania major (MHOM/IL/80/Friedlin) promastigotes and its derivative luminescent strain *L. major* LucRE9 with the luciferase gene integrated into the parasite genome (LUC) were cultured at 28 °C, 5 % CO_2 in modified RPMI-1640 medium (Invitrogen, Carlsbad, CA) supplemented with 10 % hiFBS[6], including 50 mg/ml blasticidin in the LucRE9 strain.[7]

Parasites were maintained in culture in their experimental growth phase (below 2 million *T. brucei* parasites per mL, ,10 million *L. major* parasites per mL for for promastigotes experiments), and in the stationary phase, 20 million *L. major* parasites per mL for THP-1 infection experiments.

MRC-5 cell line (human lung fibroblast) was grown in monolayer at 37 °C, 5 % CO_2 in DMEM medium (1 g/L glucose) supplemented with 10% hiFBS, 100 U/mL penicillin, 100 mg/mL streptomycin and 2 mM L-glutamine.[8]

THP-1 cells (human monocytic cell line) were maintained in RPMI-1640 medium supplemented with 10% heat-inactivated fetal bovine serum (hiFBS) and 5 % penicillin/streptomycin (37 °C, 5 % CO_2, and 100 % humidity).

Human cervical carcinoma (HeLa) and human colorectal adenocarcinoma (HT-29) cell lines were maintained at 37 °C, 5 % CO_2 in high glucose DMEM (4.5 g/L glucose) supplemented with 10% hiFBS, 100 U/ml penicillin, 100 mg/ ml streptomycin, 2 mM L-glutamine and non-essential aminoacids (1x). Cells were plated and passaged according to ATCC recommendations and were used for the experiments while in the exponential growth phase.

Fluorescence microscopy

MRC-5 cells (2×10^4 / mL) were incubated with 5 μM of compound **2b** in 0.5 mL of their respective medium for 30 min and 1 h at 37 °C and 100 % of humidity. The mitochondrial staining was performed using Mitotracker deep red (200 nM) for 30 min. Cells were washed 5 times with

room temperature PBS and fixed with paraformaldehyde 2 % for 20 min and washed two extra times with PBS. The cover slides were immerged in water and ethanol prior to sample processing for microscopy. Prolong DAPI (3–4 μL) was used as mounting medium for nuclear staining.

T. brucei parasites (2× 10^7 / mL) were incubated with 5 μM of compound **2b** in 0.5 mL of their respective medium for 30 min, 1 h and 2 h at 37 °C and 100 % humidity. The mitochondrial localization was performed using Mitotracker deep red (200 nM) for 30 min. Parasites were washed 5 times with cold PBS and fixed with paraformaldehyde 4 % for 20 min and washed two extra times with cold PBS. Prolong DAPI (3–4 μL) was used as mounting medium for nuclear staining.

Images were acquired using a widefield Olympus ix81 microscope. Excitation was done with the 385 nm, 500 nm and 633 nm filters for DAPI nuclear dye, compound **2b** and Mitotracker, respectively. Emission was detected at 450-490 nm, for DAPI nuclear dye, 602-640 nm for compound **2b** and 666-724 nm for Mitotracker. The images were processed and analyzed with Fiji software (https:// fiji.sc/).

In silico ADMET properties

The physicochemical and pharmacokinetic properties of the compounds were predicted using ADMET (absorption, distribution, metabolism, excretion and toxicity) profiles available in the ADMET lab 2.0 online server https://admetmesh.scbdd.com/ (accessed on 3 February 2025)[9], pkCSM online server https://biosig.lab.uq.edu.au/pkcsm/prediction (accessed on 20 June 2025)[10] and SwissADME online server http://www.swissadme.ch/ (accessed on 20 June 2025)[11].

1.3. Biophysical methods

FRET-melting assays

FRET melting assays were performed to assess ligand affinity for duplex and G-quadruplex DNA. Briefly, oligonucleotides of interest were obtained labelled at the 5’ and 3’ ends with FAM (a fluorescence donor) and TAMRA (a fluorescence quencher), respectively. In the folded state,

proximity of the donor and quencher result in no observed fluorescence from FAM, since energy is transferred non-radiatively to TAMRA by FRET. As the temperature is raised and the secondary structure denatures, the fluorophores move further apart and the fluorescence signal increases. From the resulting curve, the characteristic melting temperature (T_{max}, also referred to as T_m) is defined as the temperature which corresponds to the maxima of the first derivative of the normalised fluorescence signal. The change in melting temperature (ΔT_m) induced by the presence of a small molecule ligand provides an indication of the ligand's ability to stabilise the DNA structure. FRET experiments were performed according to the procedure reported by De Cian and co-workers [12] on an AriaMx qPCR instrument. The method consisted of holding at 25 °C for 5 min, before heating at 1 °C/min to 96 °C in 1 °C increments, followed by monitoring the fluorescence output at each increment for 1 min. The fluorescence emission of FAM was followed at 516 nm after excitation at 492 nm.

The final concentration of oligonucleotide was 200 nM in all cases. For F21T in Na^+ conditions, the final buffer concentration was 100 mM NaCl, and 10 mM Li Cacodylate. For FMycT (K^+ Conditions), 1 mM KCl, 99 mM LiCl and 10 mM Li Cacodylate was used. For F21T in K^+ conditions as well as for Febr1T, Fmt6363T and F10T, 10 mM KCl, 90 mM LiCl and 10 mM Li Cacodylate was used. Ligand concentrations were 10 μM. Each sample was tested in quadruplicate, and each experiment was tested in triplicate to assess the reproducibility of all results. Appropriate control experiments were also carried out for each sample set, using the ligand TmPyP4 as positive control (1 μM). Data processing was carried out using Python, with ΔT_{max} used to represent ΔT_m.

CD titrations

Circular Dichroism (CD) titrations were recorded using a Jasco J-810 spectrometer fitted with a Peltier temperature controller. Measurements were taken in a quartz cuvette with a path length of 5 mm, at 20°C, at a 50 nm / min scanning speed at 1 nm intervals, with a 1 nm bandwidth. The CD spectra were recorded between 600 and 240 nm. The reported spectrum for each sample represents the average of 3 scans and is baseline corrected for the buffer used. The

oligonucleotides were at a concentration of 5 µM in potassium phosphate buffer (100 mM, pH 7.4). Data processing was carried out using Python and Prism 10 (GraphPad Software) with a 4-point second order smoothing polynomial applied to all spectra. Observed ellipticities were converted to molar ellipticity.

UV-vis titrations

Absorbance spectra were recorded on a Cary 60 UV-Visible spectrophotometer using a room-light immune fibre optic probe with 10 mm path length. The UV-Vis spectra were recorded between 700 – 300 nm and were corrected for background and buffer absorbance.

In the titrations, the concentration of ligand was fixed at 10 µM in an initial volume of 500 µL in potassium phosphate buffer (100 mM, pH 7.4). Aliquots of oligonucleotide were added from a 100 µM stock solution containing also 10 µM ligand to maintain constant ligand concentration. Following addition, the solution was mixed thoroughly and the UV-visible spectrum was acquired immediately. Data were fitted to an independent-and-equivalent-sites binding model using Prism 10 software, a full derivation of which is provided by (amongst others) Buurma and Gade.[13] The stoichiometry of the complex (N) was set to 2/3 (ligand:G4) in order to afford satisfactory fits for the resulting isotherms. The data presented in Figure 6 shows the average values obtained from two independent experiments.

Table S1. Synthesis and photophysical data in acetonitrile solution (absorption, λ_{abs}, fluorescence emission maxima, λ_{fluo}, molar absorptivity, ε and fluorescence quantum yield, ϕ_F) for the 2-ethenyl-benzothiazolium derivatives **2a-j**.

**Compound**	**Yield (%)**	**λ_{abs} (nm)**	**Log (ε) ($M^{-1}.cm^{-1}$)**	**λ_{fluo} (nm)**	**ϕ_F**
**2a**	[2]	366; 559	4.52	575	0.083
**2b**	[2]	520	4.30	594	0.012
**2c**	[2–4]	509	4.31	-	-
**2d**	30	499	4.57	585	0.002
**2e**	[2]	465	4.38	520	0.001
**2f**	20	529	4.33	655	0.01
**2g**	[2]	559	4.40	640	0.036

**2h**	2–4	565	4.82	618	0.002
**2i**	8	529	4.56	-	-
**2j**	15	534	4.52	-	-

Table S2. Pharmacokinetic and toxicity (ADMET) properties of the compound **2b** and G4 ligands TMPyP4 and quarfloxin, according to pkCSM software.[10]

	Properties	compound 2b	TMPyP4	quarfloxin	Comment
Absorption	LogP	0.9662	10.43248	5.4892	
	Water solubility (log mol/L)	-3.647	-2.892	-4.44	
	Caco2 permeability (log Papp in 10^{-6} cm/s)	1.077	-0.913	0.453	High permeability: > 0.9
	Human Intestinal absorption (% absorbed)	81.343	7.552	100	High absorption: > 30%
	Skin Permeability (log Kp)	-2.411	-2.735	-2.735	High skin permeability: < -2.5
Distribution	Volume distribution (log L/kg)	1.171	0.008	-0.075	High Volume distribution: > 0.45
	BBB permeability (log BB)	0.447	-4.028	-0.786	High BBB permeability: > 0.3
	CNS permeability (log PS)	-1.211	-2.611	-2.127	High CNS permeability: > -2
Metabolism	CYP2D6 substrate/ inhibitor	No/Yes	No/No	No/No	
	CYP3A4 substrate/ inhibitor	Yes/No	No/No	Yes/Yes	
	CYP1A2 inhibitior	Yes	No	No	
	CYP2C19 inhibitior	Yes	No	No	
	CYP2C9 inhibitior	No	No	Yes	
Excretion	Total Clearance (log ml/min/kg)	1.134	-1.551	0.576	
Toxicity	AMES toxicity	No	No	No	
	hERG I inhibitor	No	No	No	
	hERG II inhibitor	Yes	No	Yes	
	Oral Rat Acute Toxicity (LD50, mol/kg)	1.949	2.482	2.396	
	Hepatotoxicity	No	No	No	
	Skin Sensitization	No	No	No	

Table S3. Pharmacokinetic (ADME) properties of the compound **2b** and G4 ligand Quarfloxin, according to SwissADME software.[11]

	Properties	Compound 2b	Quarfloxin
**Lipophilicity**	**Log $P_{o/w}$ (iLOGP)**	1.07	4.05
	**Log $P_{o/w}$ (XLOGP3)**	5.80	5.10
	**Log $P_{o/w}$ (WLOGP)**	0.75	5.15
	**Log $P_{o/w}$ (MLOGP)**	4.04	2.27
	**Log $P_{o/w}$ (SILICOS-IT)**	4.50	4.90
	**Consensus Log $P_{o/w}$**	3.23	4.29
**Pharmacokinetics**	**GI absorption**	High	High
	**BBB permeant**	Yes	No
	**P-gp substrate**	No	Yes
	**CYP1A2 inhibitor**	No	No
	**CYP2C19 inhibitor**	Yes	No
	**CYP2C9 inhibitor**	Yes	Yes
	**CYP2D6 inhibitor**	Yes	Yes
	**CYP3A4 inhibitor**	No	Yes
	**Log K_p (skin permeation)**	-4.76 cm/s	-6.37 cm/s
**Druglikeness / Medicinal chemistry**	**Lipinski**	Yes; 0 violation	Yes; 1 violation: MW>500
	**Bioavailability Score**	0.55	0.55
	**Leadlikeness**	No; 2 violations: MW>350, XLOGP3>3.5	No; 2 violations: MW>350, XLOGP3>3.5
	**Synthetic accessibility**	3.06	5.16

Note: TMPyP4 was excluded from this analysis due to its high molecular weight and structural complexity, which exceed the platform's input limit (maximum 200 characters per SMILES).

Table S4. G4-forming and duplex sequences included in the FRET-melting assays.

**Oligonucleotide** [14,15]	**Sequence (5'- to 3')**
**F21T -** Human telomeric G-quadruplex	FAM- GGGTTAGGGTTAGGGTTAGGG-TAMRA
**FEbr1T -** *T. brucei* G-quadruplex	FAM-GGGCAGGGGGTGATGGGGAGGAGCCAGGG-TAMRA
**FmycT -** Predominant G quadruplex formed in c-Myc Pu27 promoter	FAM- TTGAGGGTGGGTAGGGTGGGTAA-TAMRA
**Fmt6363T –** Human mitochondria G-quadruplex	FAM-AGGGACGCGGGCGGGGGATATAGGGT-TAMRA
**F10T -** Duplex DNA	FAM- TATAGCTATAHEGTATAGC-TAT-A -TAMRA

FAM = 6-carboxyfluorescein, TAMRA = 6-carboxy-tetramethylrhodamine and HEG = [(-CH_2CH_2O-)$_6$]

Table S5. Results of the FRET-melting assay performed on derivatives **2b**, **2c**, **2d**, **2g** and **2h**.

Compound	ΔT_m / °C					
	F21T K^+	F21T Na^+	FEbr1T	FMycT	Fmt6363T	F10T
**2b**	13.1 ± 0.5	9.1 ± 0.3	13.1 ± 0.6	8.6 ± 0.9	19.8 ± 0.4	2.3 ± 0.2
**2c**	12.7 ± 0.4	9.4 ± 0.5	13.5 ± 0.6	8.6 ± 1.0	10.2 ± 0.5	0.7 ± 0.1
**2d**	18.5 ± 1.9	22.9 ± 0.9	20.5 ± 1.3	16.7 ± 0.7	18.5 ± 0.9	1.0 ± 0.1
**2g**	9.7 ± 0.2	3.4 ± 0.5	8.9 ± 0.5	3.0 ± 1.1	7.7 ± 0.3	0.4 ± 0.1
**2h**	16.9 ± 0.2	9.4 ± 0.3	14.1 ± 0.5	12.6 ± 0.9	15.0 ± 0.4	0.9 ± 0.4
**ThT**	5.5 ± 0.2	1.4 ± 0.2	4.5 ± 0.7	15.0 ± 0.9	2.9 ± 0.4	0.1 ± 0.1
**TMPyP4**	16.4 ± 0.4	10.4 ± 0.4	12.4 ± 0.9	12.6 ± 1.1	7.1 ± 0.4	1.7 ± 0.1

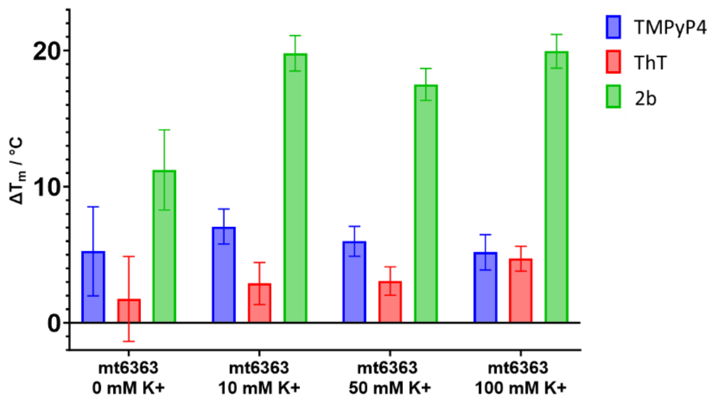

Figure S1. FRET melting analysis for compound **2b** (10 µM), ThT (10 µM) and TMPyP4 (1 µM) with mt6363 in increasing concentrations of K^+.

Table S6. G4-forming and duplex sequences included in the CD analysis.

**Oligonucleotide** [14,15]	**Sequence (5'- to 3')**
**telo23 -** hybrid type human telomeric G-quadruplex	TAG-GGT-TAG-GGT-TAG-GGT-TAG-GG
**Ebr1 -** *T. brucei* G-quadruplex	GGG-CAG-GGG-GTG-ATG-GGG-AGGAGC-CAG-GG
**mt6363 –** Human mitochondria G-quadruplex	AGGGACGCGGGCGGGGGATATAGGGT
**ds26-** self-complementary duplex	CAA-TCG-GAT-CGA-ATT-CGA-TCC-GATTG

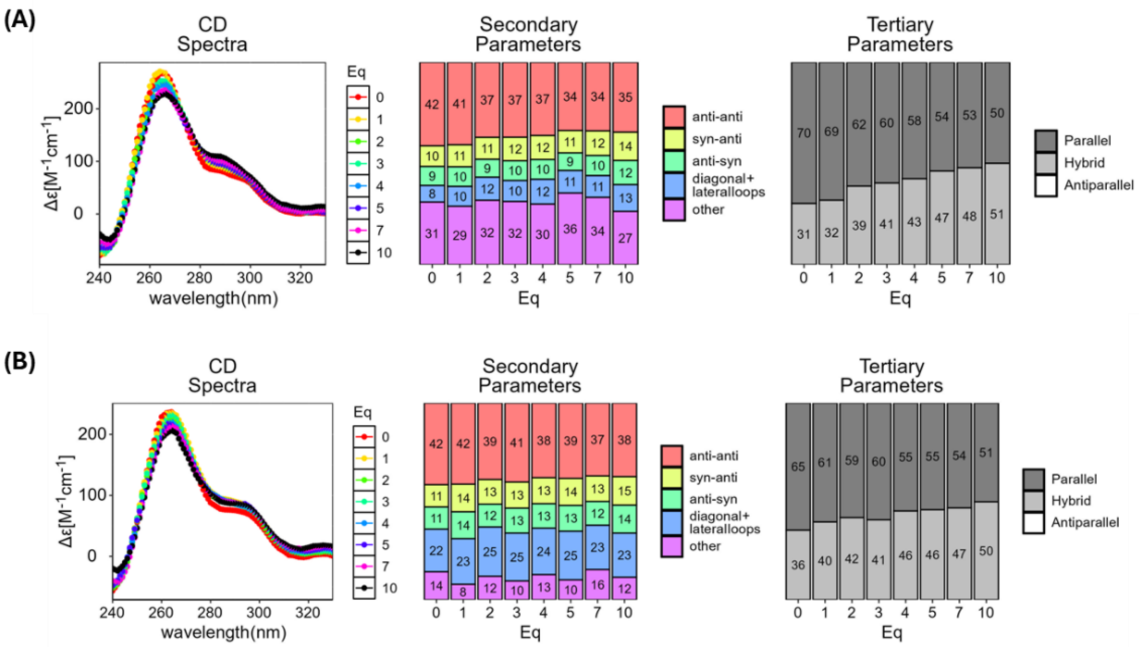

Figure S2. CD spectra of (A) mt6363 with different equivalents of compound **2b** and analysis of the influence of the number of equivalents of **2b** on the secondary and tertiary parameters; (B) Ebr1 with different equivalents of compound **2b** and analysis of the influence of the number of equivalents of **2b** on the secondary and tertiary parameters. Buffer = 100 mM potassium phosphate, pH 7.4. Oligonucleotide concentration = 5 µM.

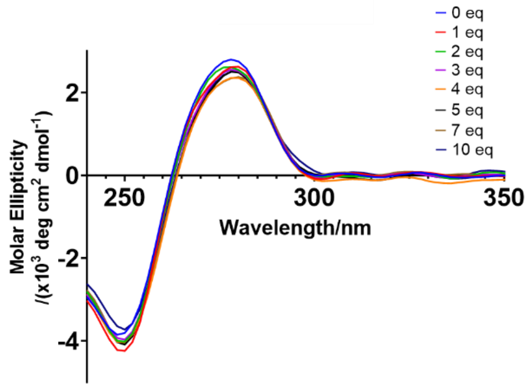

Figure S3. CD spectra of ds26 with different equivalents of compound **2b**. Buffer = 100 mM potassium phosphate, pH 7.4. Ligand concentration = 10 μM.

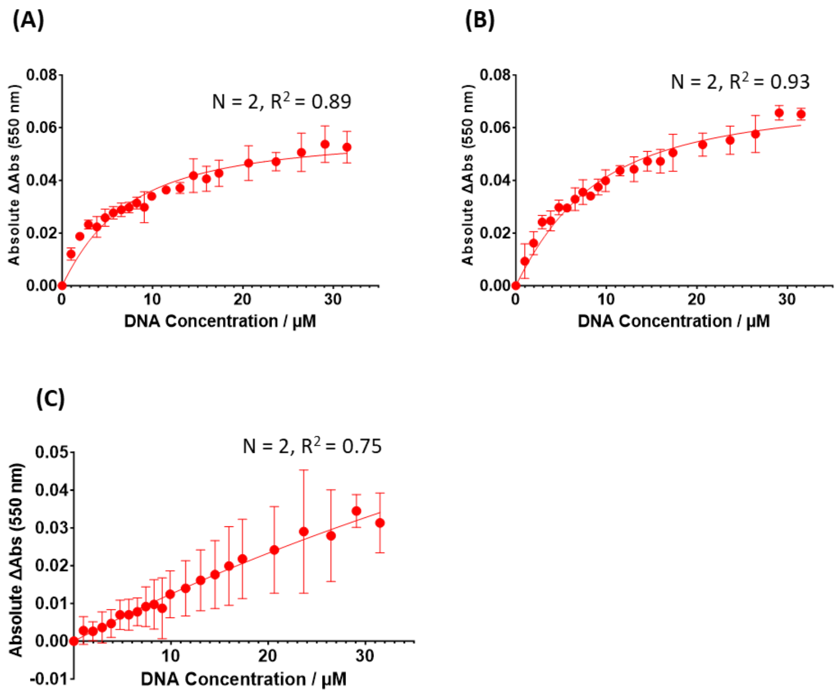

Figure S4. UV-vis analysis of compound **2c** with (A) telo23, (B) Ebr1 and (C) ds26 sequences.

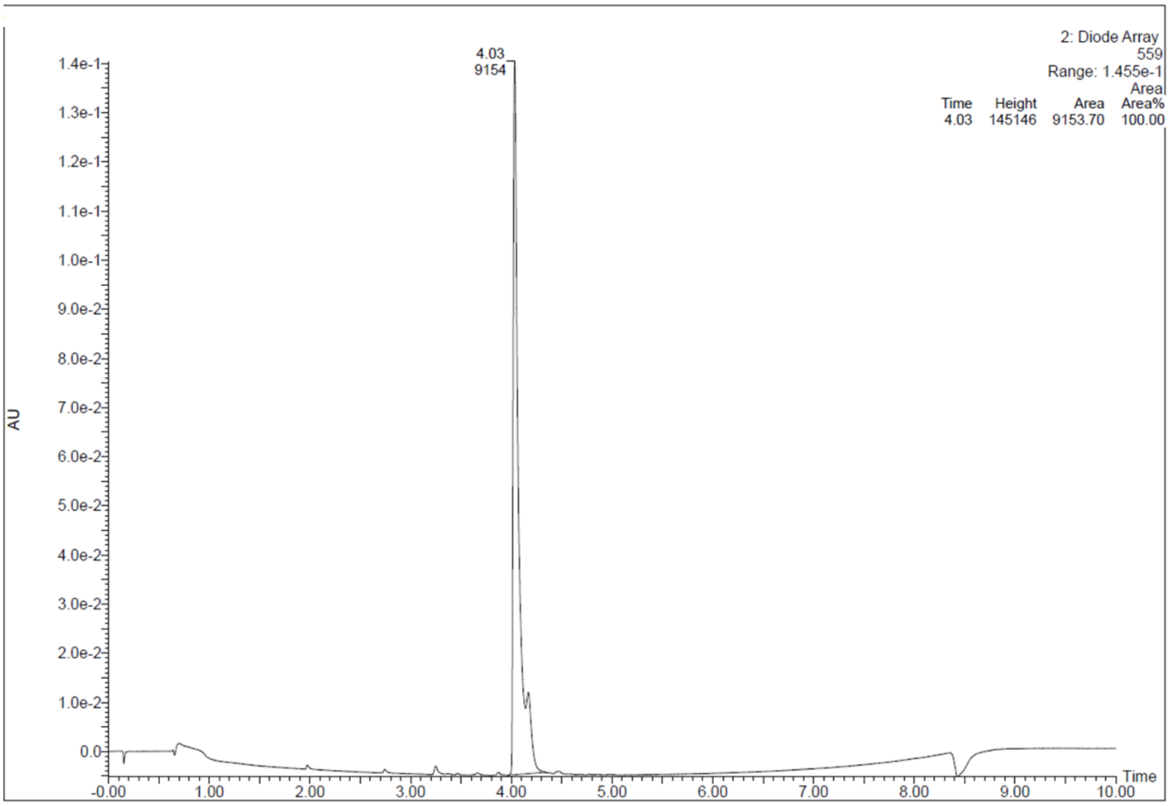

Figure S5. HPLC chromatogram of compound **2a**.

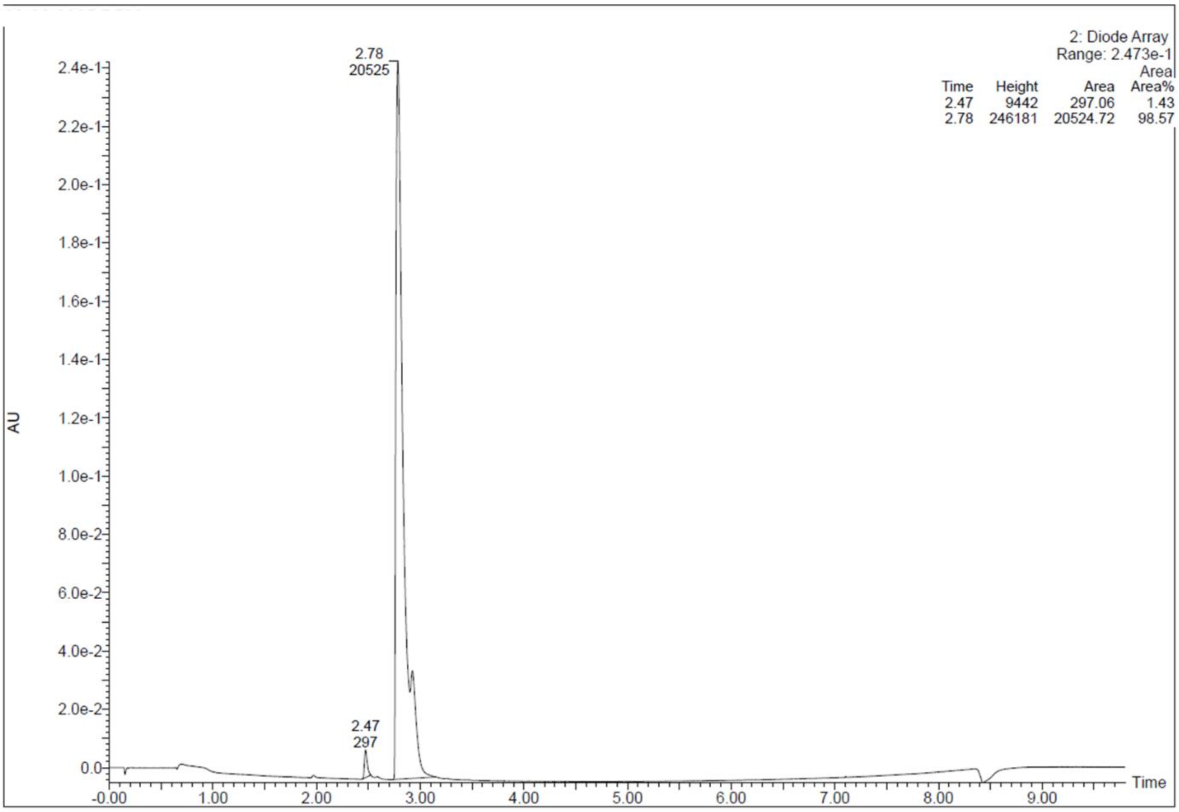

Figure S6. HPLC chromatogram of compound **2b**.

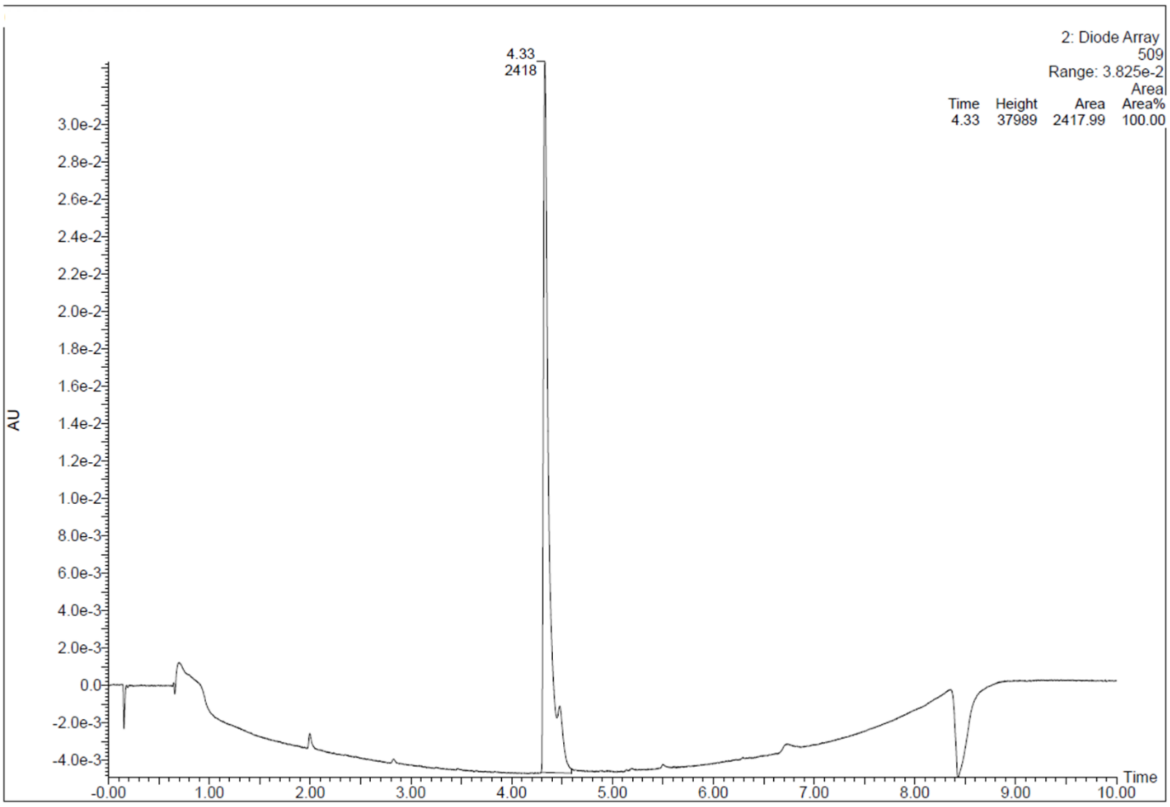

Figure S7. HPLC chromatogram of compound **2c**.

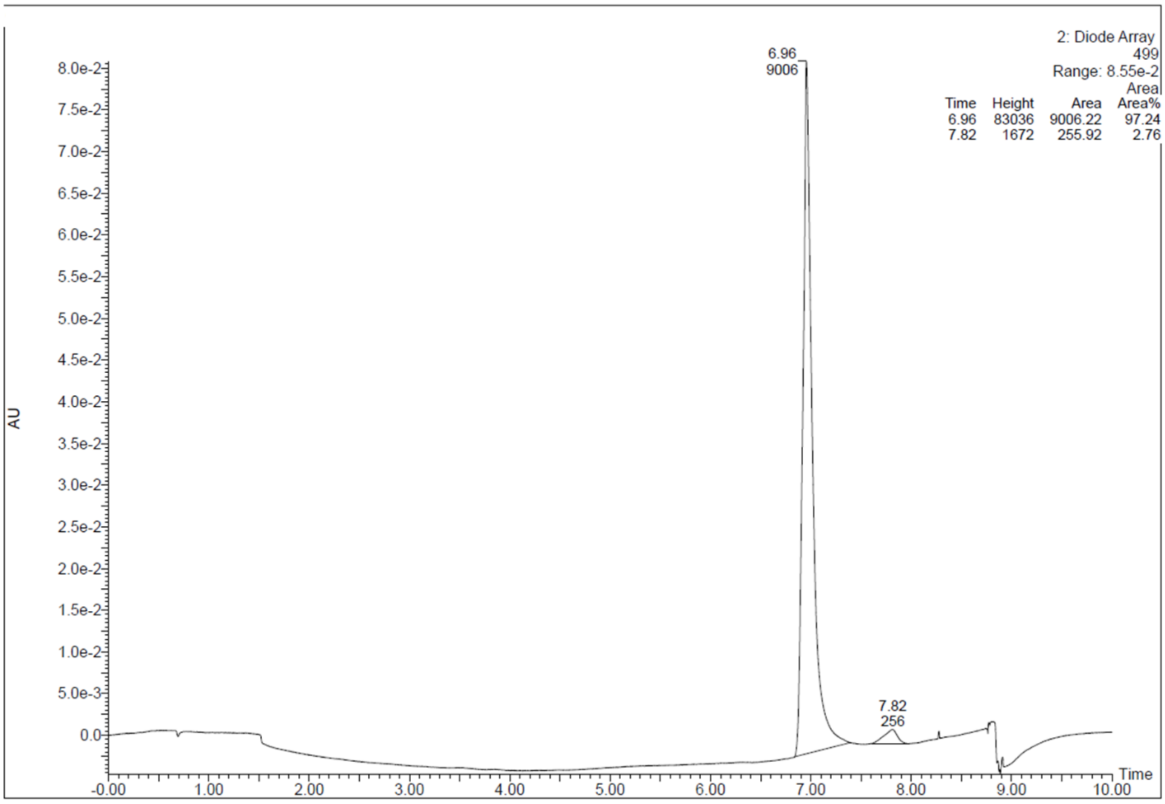

Figure S8. HPLC chromatogram of compound **2d**.

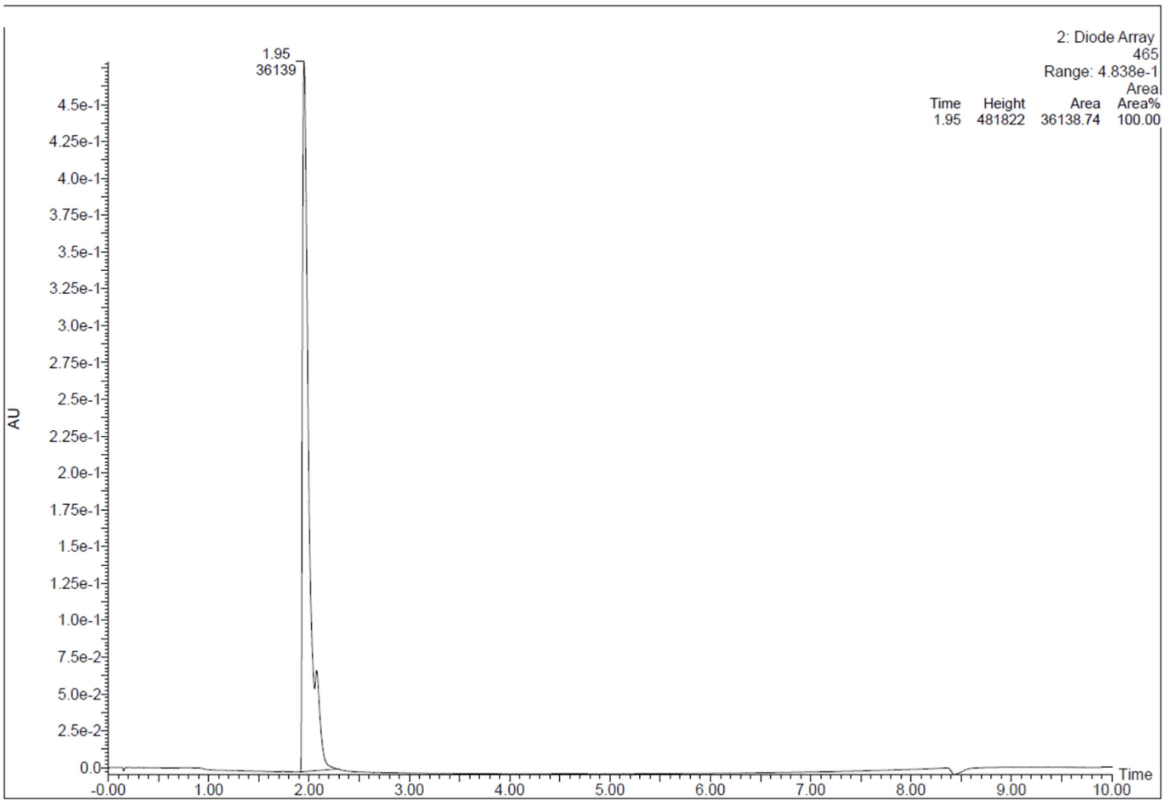

Figure S9. HPLC chromatogram of compound **2e**.

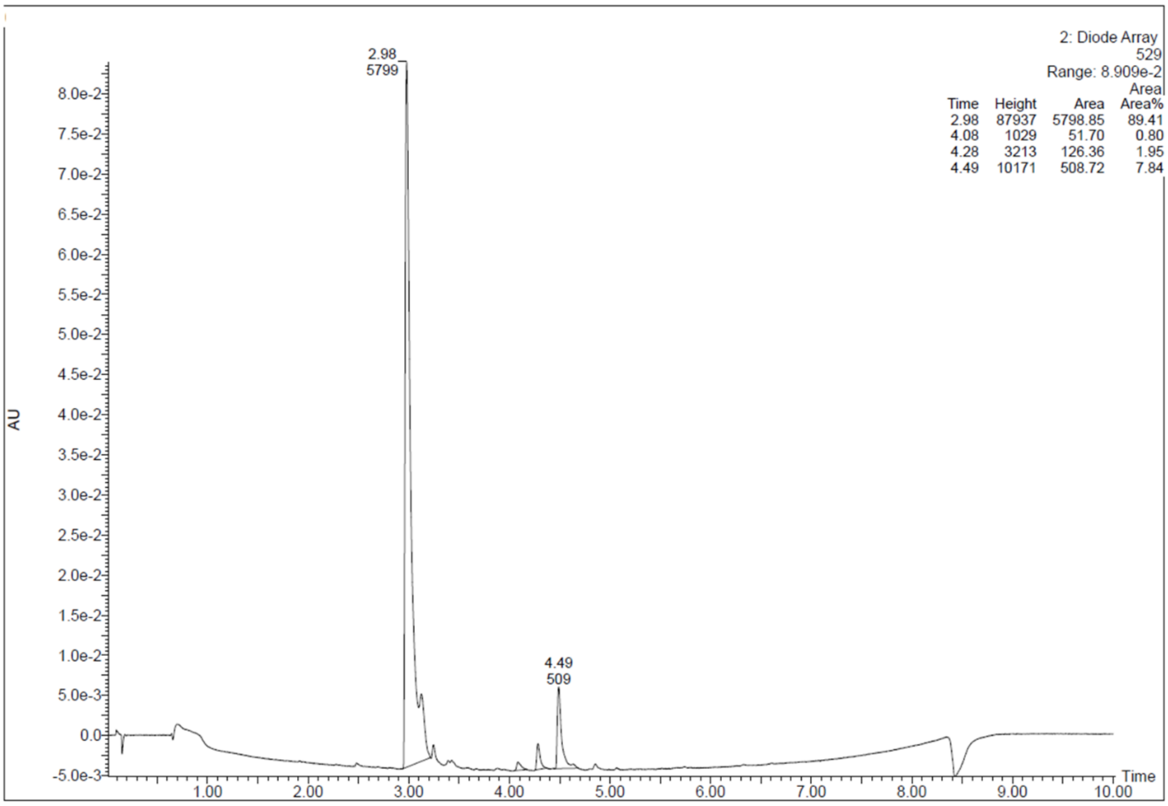

Figure S10. HPLC chromatogram of compound **2f**.

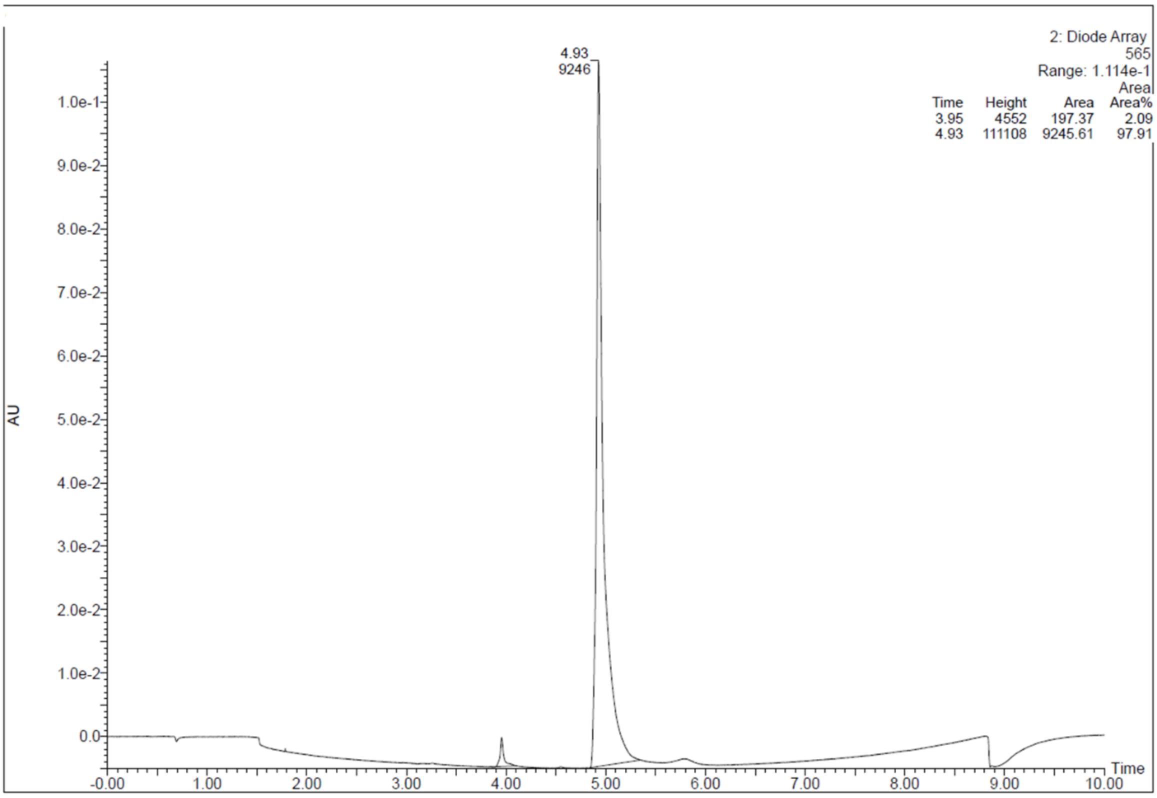

Figure S11. HPLC chromatogram of compound **2h**.

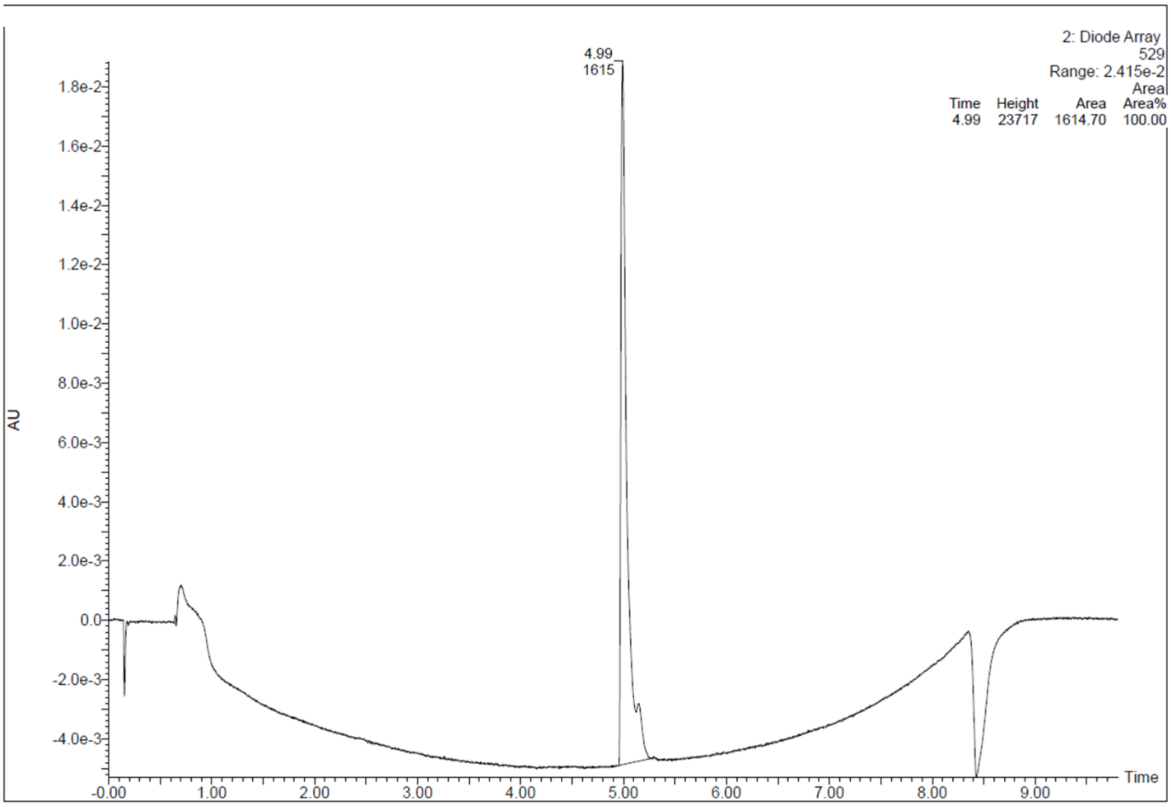

Figure S12. HPLC chromatogram of compound **2i**.

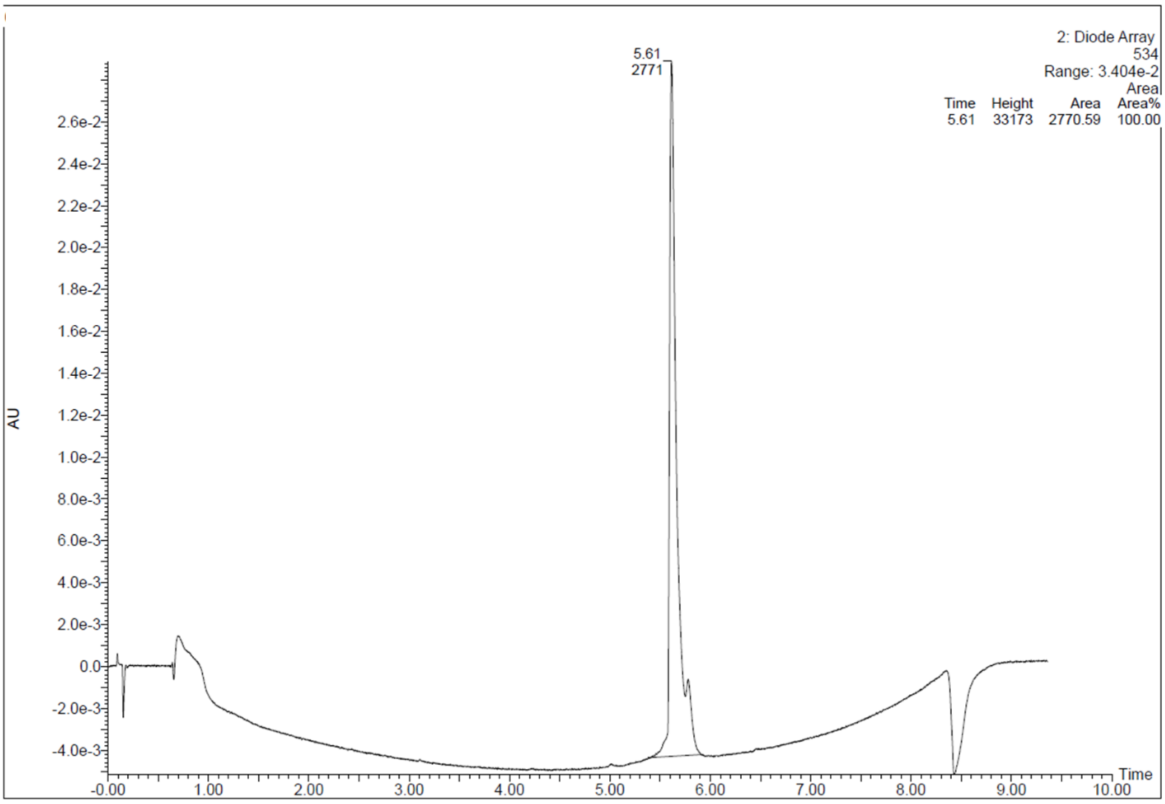

Figure S13. HPLC chromatogram of compound **2j**.

References

(1) Montalti, M.; Credi, A.; Prodi, L.; Gandolfi, M. T. *Handbook of Photochemistry*, 3rd ed.; CRC Press: Boca Raton, 2006. https://doi.org/10.1201/9781420015195.

(2) Coelho, P. J.; Castro, M. C. R.; Raposo, M. M. M. Fast (Hetero)Aryl-Benzothiazolium Ethenes Photoswitches Activated by Visible-Light at Room Temperature. *Dyes Pigm.* **2015**, *117*, 163–169. https://doi.org/10.1016/j.dyepig.2015.02.015.

(3) Hrobárik, P.; Sigmundová, I.; Zahradník, P.; Kasák, P.; Arion, V.; Franz, E.; Clays, K. Molecular Engineering of Benzothiazolium Salts with Large Quadratic Hyperpolarizabilities: Can Auxiliary Electron-Withdrawing Groups Enhance Nonlinear Optical Responses? *J. Phys. Chem. C* **2010**, *114* (50), 22289–22302. https://doi.org/10.1021/jp108623d.

(4) Fülöpová, A.; Magdolen, P.; Sigmundová, I.; Zahradník, P.; Rakovský, E.; Cigáň, M. Benzotristhiazole Based Chromophores for Nonlinear Optics. *J. Mol. Struct.* **2012**, *1027*, 70–80. https://doi.org/10.1016/j.molstruc.2012.06.018.

(5) Wirtz, E.; Leal, S.; Ochatt, C.; Cross, GeorgeA. M. A Tightly Regulated Inducible Expression System for Conditional Gene Knock-Outs and Dominant-Negative Genetics in Trypanosoma Brucei. *Mol. biochem. parasitol.* **1999**, *99* (1), 89–101. https://doi.org/10.1016/S0166-6851(99)00002-X.

(6) Cabello-Donayre, M.; Malagarie-Cazenave, S.; Campos-Salinas, J.; Gálvez, F. J.; Rodríguez-Martínez, A.; Pineda-Molina, E.; Orrego, L. M.; Martínez-García, M.; Sánchez-Cañete, M. P.; Estévez, A. M.; Pérez-Victoria, J. M. Trypanosomatid Parasites Rescue Heme from Endocytosed Hemoglobin through Lysosomal HRG Transporters: Trypanosomatid HRG Proteins Rescue Heme from Hb. *Mol. Microbiol* **2016**, *101* (6), 895–908. https://doi.org/10.1111/mmi.13430.

(7) Orrego, L. M.; Cabello-Donayre, M.; Vargas, P.; Martínez-García, M.; Sánchez, C.; Pineda-Molina, E.; Jiménez, M.; Molina, R.; Pérez-Victoria, J. M. Heme Synthesis through the Life

Cycle of the Heme Auxotrophic Parasite *Leishmania Major*. *FASEB J.* **2019**, *33* (12), 13367–13385. https://doi.org/10.1096/fj.201901274RR.
(8) Belmonte-Reche, E.; Martínez-García, M.; Guédin, A.; Zuffo, M.; Arévalo-Ruiz, M.; Doria, F.; Campos-Salinas, J.; Maynadier, M.; López-Rubio, J. J.; Freccero, M.; Mergny, J.-L.; Pérez-Victoria, J. M.; Morales, J. C. G-Quadruplex Identification in the Genome of Protozoan Parasites Points to Naphthalene Diimide Ligands as New Antiparasitic Agents. *J. Med. Chem.* **2018**, *61* (3), 1231–1240. https://doi.org/10.1021/acs.jmedchem.7b01672.
(9) Xiong, G.; Wu, Z.; Yi, J.; Fu, L.; Yang, Z.; Hsieh, C.; Yin, M.; Zeng, X.; Wu, C.; Lu, A.; Chen, X.; Hou, T.; Cao, D. ADMETlab 2.0: An Integrated Online Platform for Accurate and Comprehensive Predictions of ADMET Properties. *Nucleic Acids Res.* **2021**, *49* (W1), W5–W14. https://doi.org/10.1093/nar/gkab255.
(10) Pires, D. E. V.; Blundell, T. L.; Ascher, D. B. pkCSM: Predicting Small-Molecule Pharmacokinetic and Toxicity Properties Using Graph-Based Signatures. *J. Med. Chem.* **2015**, *58* (9), 4066–4072. https://doi.org/10.1021/acs.jmedchem.5b00104.
(11) Daina, A.; Michielin, O.; Zoete, V. SwissADME: A Free Web Tool to Evaluate Pharmacokinetics, Drug-Likeness and Medicinal Chemistry Friendliness of Small Molecules. *Sci Rep* **2017**, *7* (1). https://doi.org/10.1038/srep42717.
(12) Decian, A.; Guittat, L.; Kaiser, M.; Sacca, B.; Amrane, S.; Bourdoncle, A.; Alberti, P.; Teuladefichou, M.; Lacroix, L.; Mergny, J. Fluorescence-Based Melting Assays for Studying Quadruplex Ligands. *Methods* **2007**, *42* (2), 183–195. https://doi.org/10.1016/j.ymeth.2006.10.004.
(13) Hahn, L.; Buurma, N. J.; Gade, L. H. A Water-Soluble Tetraazaperopyrene Dye as Strong G-Quadruplex DNA Binder. *Chem. Eur. J.* **2016**, *22* (18), 6314–6322. https://doi.org/10.1002/chem.201504934.
(14) Street, S. T. G.; Peñalver, P.; O'Hagan, M. P.; Hollingworth, G. J.; Morales, J. C.; Galan, M. C. Imide Condensation as a Strategy for the Synthesis of Core-Diversified G-Quadruplex Ligands with Anticancer and Antiparasitic Activity**. *Chem. Eur. J.* **2021**, *27* (28), 7712–7721. https://doi.org/10.1002/chem.202100040.
(15) Huang, W.-C.; Tseng, T.-Y.; Chen, Y.-T.; Chang, C.-C.; Wang, Z.-F.; Wang, C.-L.; Hsu, T.-N.; Li, P.-T.; Chen, C.-T.; Lin, J.-J.; Lou, P.-J.; Chang, T.-C. Direct Evidence of Mitochondrial G-Quadruplex DNA by Using Fluorescent Anti-Cancer Agents. *Nucleic Acids Res.* **2015**, gkv1061. https://doi.org/10.1093/nar/gkv1061.
